# Supplementary material for: Volcanic forcing degrades multiyear-to-decadal prediction skill in the tropical Pacific
Source: Sci Adv. 2023 Apr 12;9(15):eadd9364. doi: 10.1126/sciadv.add9364 (PMC10096591; doi:10.1126/sciadv.add9364)
Supplement: 20230412-1 [file sciadv.add9364.v1.pdf]

CLIMATOLOGY

Copyright © 2023 The Authors, some rights reserved; exclusive licensee American Association for the Advancement of Science. No claim to original U.S. Government Works. Distributed under a Creative Commons Attribution License 4.0 (CC BY).

Volcanic forcing degrades multiyear-to-decadal prediction skill in the tropical Pacific

Xian Wu*, Stephen G. Yeager, Clara Deser, Nan Rosenbloom, Gerald A. Meehl

Volcanic aerosol forcing can affect global climate, but its role in climate prediction remains poorly understood. We isolate the impact of volcanic eruptions on multiyear-to-decadal climate prediction skill by comparing two suites of initialized decadal hindcasts conducted with and without historical volcanic forcing. Unexpectedly, the inclusion of volcanic forcing in the prediction system significantly degrades the forecast skill of detrended multiyear-to-decadal sea surface temperature (SST) variability in the central-eastern tropical Pacific. The ensemble mean hindcasts produce multiyear-to-decadal tropical Pacific SST cooling in response to large tropical volcanic eruptions through thermodynamic and El Niño–Southern Oscillation (ENSO)–like dynamic processes. However, in observations, these eruptions coincided with tropical Pacific warming, which is well predicted by the no-volcano hindcasts and, hence, is likely related to internal climate variability. Improved model representation of volcanic response and its interaction with internal climate variability is required to advance prediction of tropical Pacific decadal variability and associated global impacts.

INTRODUCTION

Large volcanic eruptions affect the global climate system by injecting sulfur gases into the stratosphere, where they are converted into stratospheric sulfate aerosols that modulate atmospheric radiative and dynamical processes (*1*, *2*). The volcanic influence has been detected in a wide range of atmosphere (*3*–*6*), ocean (*7*–*10*), land, and sea ice variables (*8*, *9*) on seasonal-to-multidecadal time scales. These volcanic impacts can be obscured by internal climate variability (*11*) and are often challenging to isolate because of the limited number of large volcanic eruptions over short modern instrumental records (since ~1850) and the lack of measurements of stratospheric volcanic sulfate aerosol before the satellite era (*1*). For example, the global mean surface temperature (GMST) cooling, one of the most prominent climate responses to volcanic eruptions, can be masked by the warming effect of El Niño events or amplified by the cooling effect of La Niña events that happen to occur as part of the intrinsic El Niño–Southern Oscillation (ENSO) phenomenon (*12*–*16*). Paleoclimate data and climate model experiments have been widely used to deduce statistically significant volcanic impacts, but there are still many sources of uncertainty that cloud our understanding of the climate response to volcanoes. These include, but are not limited to, large disagreements among different paleoclimate reconstructions (*17*, *18*), model disagreements and deficiencies in simulating volcanic responses (*4*, *19*–*22*), and uncertainties in estimating the observed volcanic forcing (*23*, *24*).

Given the hypothesized strong climate forcing, volcanic eruptions could provide an important source of predictability for post-eruption climate evolution. Historical volcanic aerosol forcing has been incorporated into the Coupled Model Intercomparison Project 5/6 (CMIP5/6) initialized decadal hindcasts to facilitate direct comparison with uninitialized historical simulations (*25*, *26*). We note that, in practice, volcanic eruptions cannot be reliably predicted in advance, and this increases the uncertainty of real-time predictions and future climate projections (*5*, *27*). To isolate the impact of volcanic eruptions on decadal predictions, the CMIP6 Decadal Climate Prediction Project protocol included an experiment to repeat hindcasts initialized in 1963, 1982, and 1991 without the aerosol forcing from the Agung, El Chichón, and Pinatubo eruptions, respectively (*26*). By comparing the predictions with and without volcanic forcing for these selected dates, a recent study (*28*) showed that volcanic forcing can robustly affect the predictions of many climate variability modes. However, relating the predicted volcanic response to observations is complicated by model deficiencies in simulating volcanic responses and/or by the presence of internal variability in nature. For example, the decadal prediction skill of CMIP5 models for tropical Pacific sea surface temperature (SST) anomalies was hypothesized to be decreased by the Pinatubo forcing because of the discrepancy between the multimodel mean volcanic response and strong internal variability in observations (*29*).

Except for a few case studies (*28*–*30*), it remains largely unexplored and unclear how volcanic forcing interacts with internal climate variability to modify the prediction skill of multiyear-to-decadal climate variability. In the present study, we investigate the volcanic effect on near-term (annual-to-decadal time scale) predictions by comparing the Community Earth System Model version 1 (CESM1) Decadal Prediction Large Ensemble (DPLE) (*31*) with a parallel set that excludes historical volcanic forcing (DPLE_NoVolc) over the period 1954–2015 (see Materials and Methods). This comparison allows for a comprehensive assessment of the influence of large volcanic eruptions on multiyear-to-decadal prediction skill over the past 60 years, including skill dependence on lead time and background conditions. The ensemble prediction framework offers a unique perspective for examining the realism of simulated volcanic impacts and unraveling the factors contributing to discrepancies between model and observations.

Climate and Global Dynamics Division, National Center for Atmospheric Research, Boulder, CO, USA.
*Corresponding author. Email: xianwu@ucar.edu

RESULTS

Volcanic impacts on the multiyear-to-decadal prediction skill of CESM1

We assess the volcanic effect on the pentadal to decadal prediction skill of global SST and land surface air temperature (SAT) by comparing the anomaly correlation coefficient (ACC) of DPLE with the ACC of DPLE_NoVolc at forecast years 1 to 5 (FY1–5), FY6–10, and FY1–10 over the period 1954–2015 (Fig. 1, A to I). Here, we focus on predictions of quadratically detrended surface temperature time series to highlight skill at predicting variations about the long-term background anthropogenic climate change signal. Results remain very similar if we use linear detrending to remove forced climate change (fig. S1), while removing the ensemble mean of uninitialized simulations at each time step will also remove the volcanic response and is not a preferred method here (fig. S2 and Materials and Methods). The high ACCs for non-detrended data show little difference between DPLE and DPLE_NoVolc nearly everywhere, as they are dominated by the anthropogenic trend (fig. S3). DPLE and DPLE_NoVolc show statistically significant ACC differences at the 90% confidence level (Materials and Methods) for detrended surface temperature over many regions (Fig. 1, A to I). In particular, the ACC is degraded in DPLE compared to DPLE_NoVolc over the central tropical Pacific and along the west coast of Mexico by up to ~0.3 for FY1–5 (Fig. 1G), ~0.8 for FY6–10

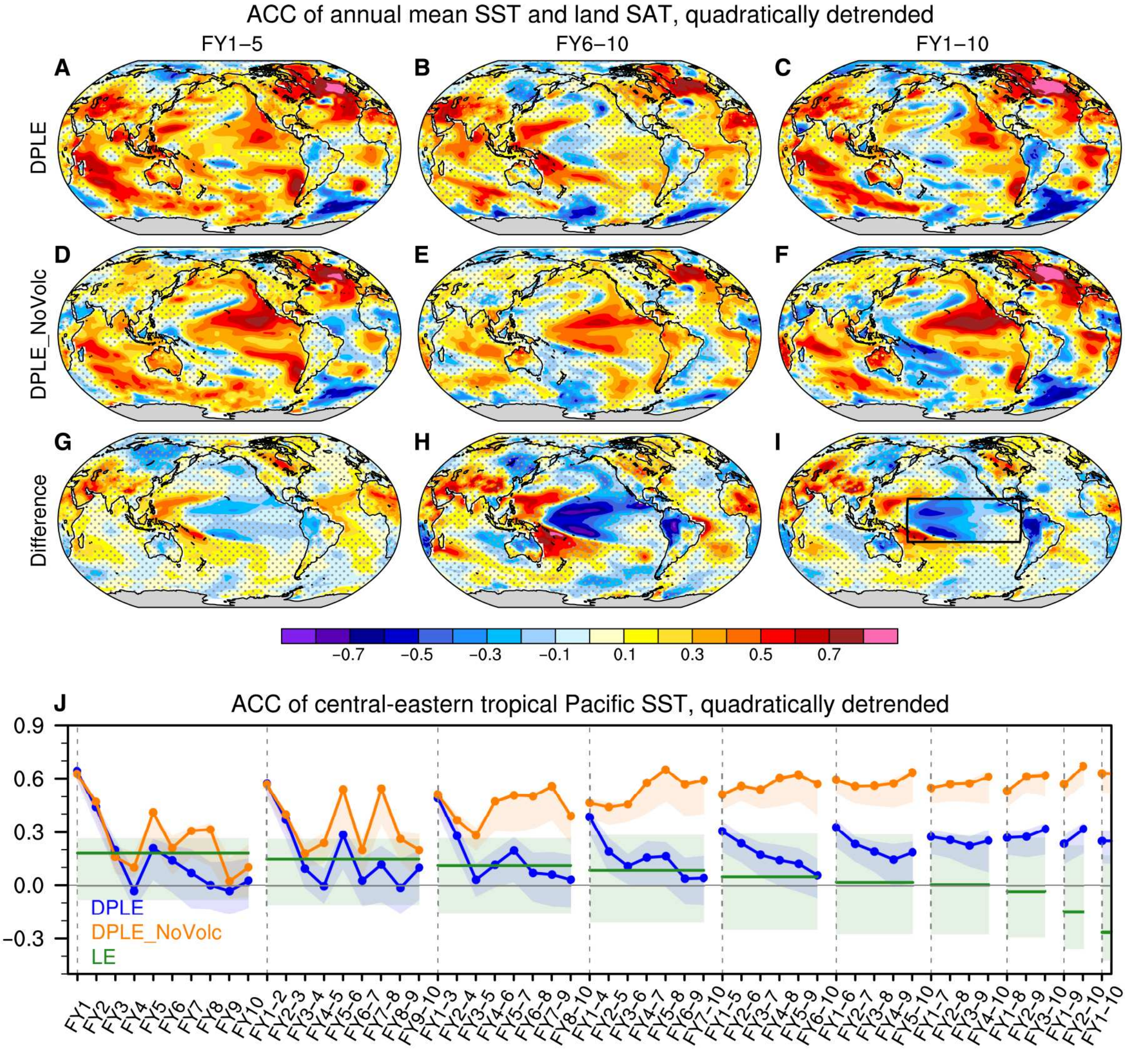

Fig. 1. Volcanic impact on the prediction skill of quadratically detrended multiyear-to-decadal surface temperatures. Anomaly correlation coefficient (ACC)of quadratically detrended annual sea surface temperature (SST) and surface air temperature (SAT) over land at FY1–5, FY6–10, and FY1–10 (from left to right columns) during 1955–2015 for (**A** to **C**) DPLE, (**D** to **F**) Decadal Prediction Large Ensemble (DPLE)_NoVolc [verified against the Extended Reconstruction Sea Surface Temperature version 5 (ERSSTv5) and Berkeley Earth Surface Temperature data], and (**G** to **I**) their difference (DPLE minus DPLE_NoVolc). The stippling indicates insignificant values at the 90% confidence level based on bootstrapping across both time and ensemble members (see Materials and Methods). (**J**) ACC of quadratically detrended central-eastern tropical Pacific SST [20°S to 20°N, 160°E to 80°W; the region is denoted by the black box in (I)] as a function of lead time intervals corresponding to 1- to 10-year averages for DPLE (blue curves), DPLE_NoVolc (orange curves), and Large Ensemble (LE) (green lines). Colored shading denotes the 10th to 90th percentile ranges of ACC based on 5000 bootstrapped ensemble means from randomly sampled 10-member ensembles, while the curves show ACC for the full ensembles (40, 10, and 40 members for DPLE, DPLE_NoVolc, and LE, respectively).

(Fig. 1H), and ~0.6 for FY1–10 (Fig. 1I). In contrast, the ACC is improved over the western tropical Pacific at all three lead times when volcanic forcing is included. DPLE_NoVolc shows a high skill (ACC > 0.5) in predicting detrended pentadal SST anomalies over 0 to 20°N in the tropical Pacific and positive ACC in the central-eastern tropical Pacific (Fig. 1, D to F), a region that has stood out as showing low skill in CMIP5/6 decadal prediction systems (*32*, *33*). In addition to the tropical Pacific, volcanic forcing significantly affects the ACC of detrended SAT over many land regions, including South Asia, tropical Africa, South America, and South Africa (Fig. 1, G to I), but the magnitude and significance of these influences vary with lead time. The rest of the paper will focus on the volcanic influence on tropical Pacific SST prediction skill.

The sensitivities to lead time and temporal averaging are explored by comparing the ACC for detrended SST anomalies averaged over the central-eastern tropical Pacific (20°S to 20°N, 160°E to 80°W) as a function of lead time and averaging interval (Fig. 1J). We focus on the central-eastern tropical Pacific to encompass the area that is not only significantly influenced by volcanic forcing (Fig. 1, G to I) but also shows positive correlation skill in the DPLE_NoVolc (Fig. 1, D to F). The results remain similar if we use a smaller region over the central tropical Pacific, which shows the strongest volcanic influence (fig. S4B). DPLE shows either comparable or significantly lower ACC than DPLE_NoVolc for all lead times and averaging intervals, but the magnitude of skill difference depends on these verification choices. For 1- and 2-year averages close to initialization (e.g., FY1, FY2, FY1 and FY2, and FY1–3), DPLE and DPLE_NoVolc show very comparable ACC scores, because the prediction skill for these lead times is largely controlled by initialization rather than by external forcing (fig. S5). For 3- to 5-year sliding averages, the differences between DPLE_NoVolc and DPLE increase with increasing lead time and become most pronounced at long lead times (e.g., cf. FY1–3 and FY8–10). The large skill differences at FY8–10, FY7–10, and FY6–10 are largely due to the decreased skill in DPLE at longer lead times, which becomes comparable to the uninitialized CESM1 Large Ensemble (LE) (see Materials and Methods) (*34*), potentially because of a loss of initialization memory and stronger volcanic influence at longer lead times. A slight increase of ACC with lead time is found for DPLE_NoVolc, but this is not statistically significant for 5-year sliding averages (fig. S6, F to J). Such increase of prediction skill with lead time in both DPLE and DPLE_NoVolc is found to be significant for non-detrended data in the southeast tropical Pacific for 5-year sliding averages (fig. S7), which is speculated to be related to the initialization shock and spurious ENSO conditions that degrade the skill at short lead times (*31*). Further analysis is needed to understand these lead time–dependent features of ACC. In the next two sections, we will investigate the mechanisms by which the volcanic forcing affects the prediction skill in the tropical Pacific.

Tropical Pacific SST response to major volcanic eruptions

To understand how the volcanic forcing degrades prediction skill, we first compare the time series of detrended central-eastern tropical Pacific SST anomalies in observations and forecasts for three different lead time windows (Fig. 2, A to C). Statistically significant differences between the ensemble mean time series from DPLE and DPLE_NoVolc tend to occur in the forecasts that overlap the strong volcanic eruptions, namely, Agung (1963), El Chichón (1982), and Pinatubo (1991) (Fig. 2D). In response to these strong volcanic eruptions, the detrended pentadal (FY1–5 and FY6–10) and decadal (FY1–10) tropical Pacific SST anomalies tend to be lower in DPLE than in DPLE_NoVolc and deviate more from the positive SST anomalies in observations. In contrast, DPLE exhibits higher tropical Pacific SST than DPLE_NoVolc in forecasts with start years of around 1970. These SST differences are not directly related to the two small volcanic eruptions in 1969 and 1974 but are, instead, artifacts of the cumulative effect of volcanic forcing on model climatology and trend (figs. S8 and S9). The annual climatology of DPLE is cooler than DPLE_NoVolc for each forecast year (fig. S9A), so the drift correction artificially makes de-drifted SST anomalies in DPLE warmer than in DPLE_NoVolc starting around 1970 when there is no significant volcanic effect on the original SSTs (fig. S8; see Supplementary Text for more details). The forecasts at FY6–10 and FY1–10 are influenced more by strong eruptions than those at FY1–5 (denoted by triangles at the bottom of Fig. 2, A to C) because, for example, volcanic forcing could happen either during FY6–10 or before FY6–10 to affect FY6–10 anomalies. This cumulative influence of volcanic forcing contributes to the more severe skill degradation for longer lead times (e.g., cf. FY1–5 and FY6–10) and averaging windows (e.g., cf. FY1–5 and FY1–10), as does the memory loss effect that increases the relative influence of external forcing over initialization (Fig. 1J). This lead time dependence of the volcanic effect on predictions would not be evident in sensitivity experiments that target only a few initialization dates (*26*).

Is the large post-eruption discrepancy between observations and DPLE caused by model error in simulating the climate response to volcanic forcing, underestimated predictable internal variability in model compared to observations, or a combination of the two? We compare the single observed realization of detrended central-eastern tropical Pacific SST anomalies during 1991–1995 following the Pinatubo eruption to the ensemble distributions from DPLE, DPLE_NoVolc, and LE (Fig. 2, E and F). The observed pentadal SST anomaly (0.2°C; vertical black line) falls within the tail of the distribution of the 40-member DPLE at FY1–5 (blue shading), while the 10-member DPLE_NoVolc distribution (orange shading) is shifted toward the observed value. The distributions of DPLE and DPLE_NoVolc are significantly different with high confidence ($P < 0.01$) based on z statistics ($z = 7.95$ for FY1–5 and $z = 3.37$ for FY6–10). The comparison between DPLE and DPLE_NoVolc shows that volcanic forcing induces a cooling that makes the observed anomaly a very unlikely outcome in DPLE. This suggests several possible explanations for a low DPLE skill: (i) The cooling response to volcanic forcing in DPLE is too strong, (ii) the predictable internal variability arising from initialization in DPLE is too weak, or (iii) the observed pentadal SST anomalies are dominated by unpredictable internal variability (e.g., warm anomaly in 1991–1995 was an unpredictable event). The relatively high overall skill from DPLE_NoVolc (ACC > 0.5) and better match to observations in the early 1990s (at both FY1–5 and FY6–10) suggest that internal variability related to slow oceanic processes was predictable to some extent. In DPLE_NoVolc, the ratio of predictable component (RPC) ~ 1 suggests that the predictable component of variance is realistic and there is no strong evidence of a signal-to-noise paradox (Materials and Methods). However, RPC computed from a 10-member ensemble (DPLE_NoVolc) may have large error bars

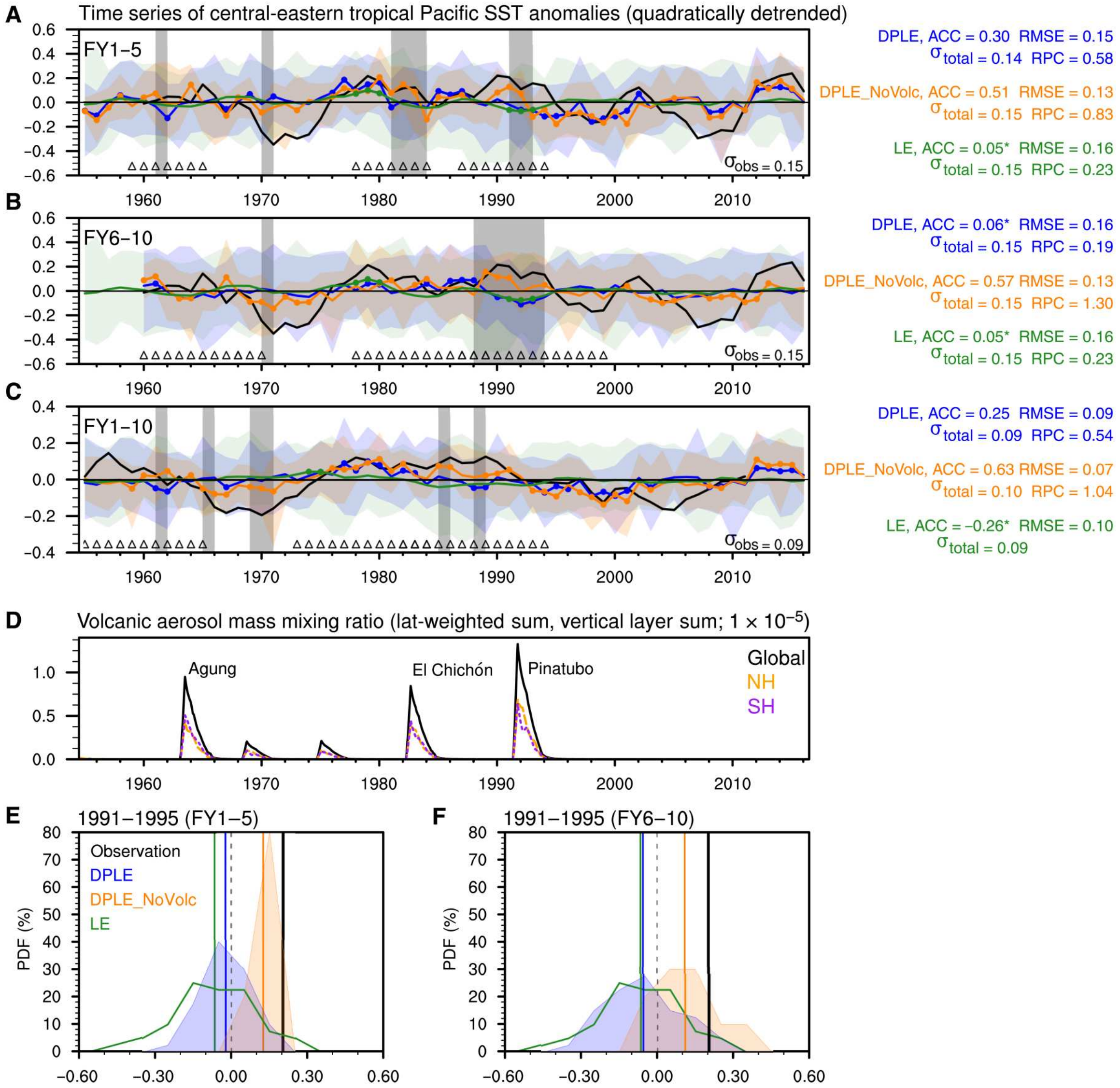

Fig. 2. Tropical Pacific sea surface temperature (SST) anomalies following major tropical volcanic eruptions. Time series of quadratically detrended SST (°C) anomalies (curves) over the central-eastern tropical Pacific (20°S to 20°N, 160°E to 80°W) in observations (black) and ensemble mean forecasts/simulations from Decadal Prediction Large Ensemble (DPLE) (blue), DPLE_NoVolc (orange), and Large Ensemble (LE) (green) for (**A**) FY1–5, (**B**) FY6–10, and (**C**) FY1–10. Shading denotes the range (minimum to maximum) of 40, 10, and 40 members for DPLE, DPLE_NoVolc, and LE, respectively. The year value in the *x* axis denotes the start year of any 5- or 10-year averaging window [e.g., 1960 represents 5-year average spanning 1960–1964 corresponding to November 1959 initialization for FY1–5 in (A) and November 1954 initialization for FY6–10 in (B)]. The triangles at the bottom of (A) to (C) denote the forecast ensembles that coincide with any of the three volcanic eruptions starting in 1963, 1982, and 1991. The colored dots on curves denote the ensemble mean anomalies that are significant at the 90% confidence level, and the gray blocks highlight time periods when the ensemble-mean difference between DPLE and DPLE_NoVolc is significant at the 90% confidence level based on bootstrapping across ensemble members (Materials and Methods). The standard deviation (SD) of observed SST time series is indicated at the bottom right of each panel. The anomaly correlation coefficient (ACC) (* denotes insignificant values), root mean square error (RMSE), SD of total variability, and ratio of predictable component (RPC) for the forecasts/simulations are indicated on the right of each panel. (**D**) Monthly time series of volcanic aerosol mass mixing ratios (curves) used in DPLE and LE for the global (black), Northern Hemisphere (NH; orange), and Southern Hemisphere (SH; purple) averages. Probability distribution functions (PDF; %) of the central-eastern tropical Pacific SST anomalies during 1991–1995 in individual members of DPLE (blue shading; 40 members), DPLE_NoVolc (orange shading; 10 members), and LE (green curves; 40 members) at (**E**) FY1–5 and (**F**) FY6–10, with vertical lines denoting the ensemble mean and observed values.

and would likely increase with a larger ensemble (see discussion in Materials and Methods). The total variance (σ^2_{total}) of tropical Pacific SST anomalies is comparable between model and observations, while PVF ($\sigma^2_{signal}/\sigma^2_{total}$) is smaller in DPLE than DPLE_NoVolc [values are not shown in Fig. 2 but can be calculated using (ACC/RPC)], suggesting that volcanic forcing may suppress the signal variance but exacerbate the noise in forecasts. Lower ACC scores in DPLE compared to DPLE_NoVolc suggests that the forced response to volcanic forcing in DPLE overwhelms the predictable internal signal. At FY6–10, both the ensemble mean and distribution of individual members of DPLE become very comparable to LE (green curve), which confirms the memory loss of initial conditions and is consistent with the ACC analysis shown in Fig. 1J.

We next focus on the periods following the strong volcanic eruptions and select three pentads (1961–1965, 1982–1986, and 1991–1995) during which the tropical Pacific SST predictions are significantly influenced by the major volcanic eruptions at FY1–5 (Fig. 2A) and FY6–10 (Fig. 2B). Figure 3 shows the spatial patterns of global detrended pentadal surface temperature anomalies for these time periods in observations and forecasts. Observations show positive SST anomalies over the central or eastern tropical Pacific but negative anomalies over the western tropical Pacific for all three pentads (Fig. 3, A to C; note that Fig. 3D replicates Fig. 3C). The observed central or eastern tropical Pacific SST warming is captured by DPLE_NoVolc (Fig. 3I-L) but not by DPLE (Fig. 3, E to H) because of the cooling effect caused by volcanic forcing in the ensemble mean hindcasts (Fig. 3, M to P). The western tropical Pacific SST cooling, on the other hand, is better reproduced when the volcanic forcing is included, which partly explains the increased prediction skill over the western tropical Pacific in DPLE than DPLE_NoVolc (Fig. 1, G to I). The increased skill in DPLE over the western tropical Pacific is also related to the excessive westward extension of positive SST anomalies in DPLE_NoVolc, which is likely associated with the ENSO pattern bias in CESM1 and ultimately caused by the mean-state equatorial Pacific cold tongue bias (*35*, *36*). Despite the SST pattern bias in the western tropical

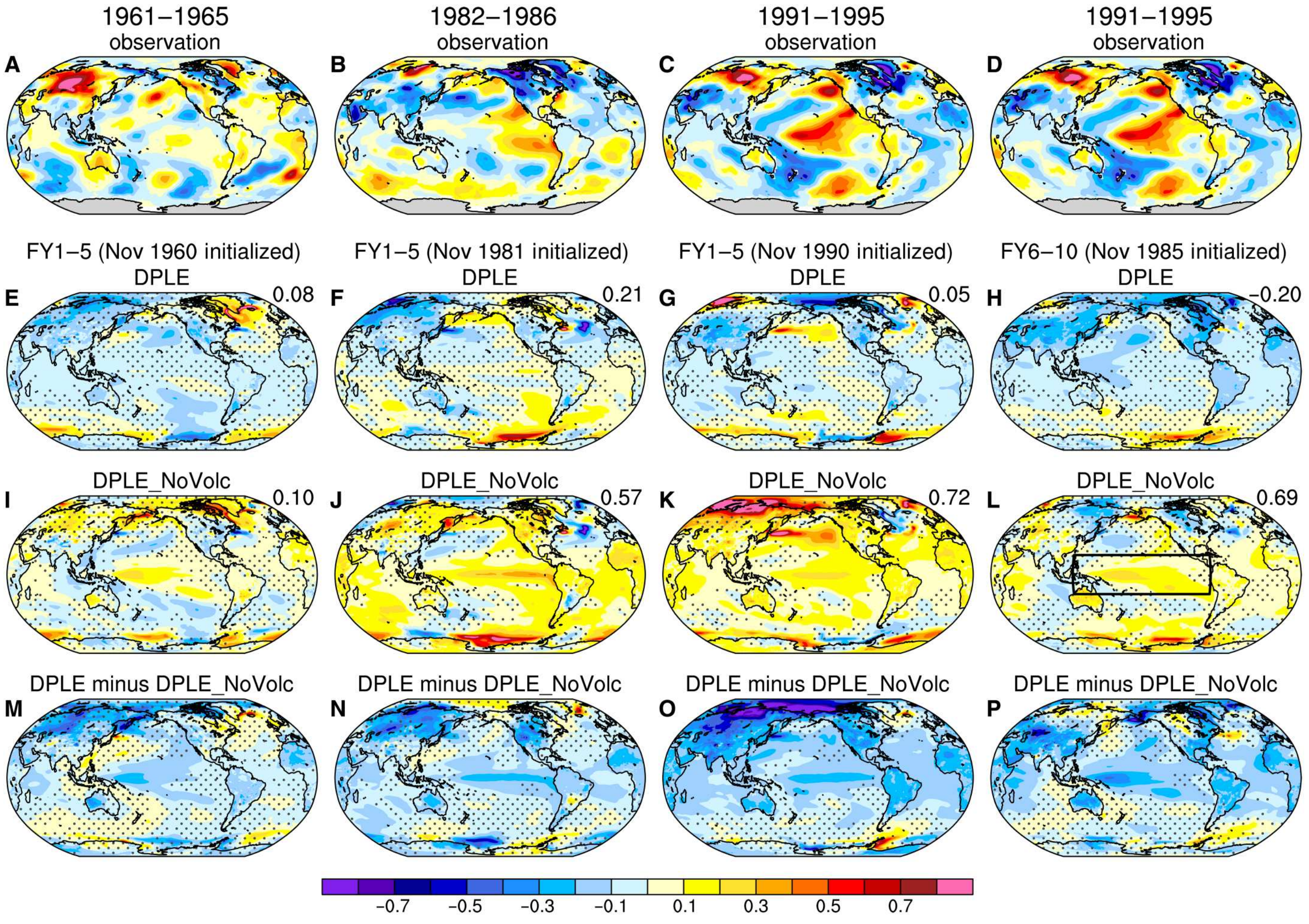

Fig. 3. Global surface temperature anomalies following the major volcanic eruptions. Five-year average quadratically detrended global sea surface temperature (SST) and land surface air temperature (SAT) (°C; color shading) anomalies during (first column) 1961–1965, (second column) 1982–1986, and (third and fourth columns) 1991–1995 in (**A** to **D**) observations; the ensemble mean forecasts of (**E** to **H**) Decadal Prediction Large Ensemble (DPLE), (**I** to **L**) DPLE_NoVolc, and (**M** to **P**) the difference between DPLE and DPLE_NoVolc at FY1–5 or FY6–10. The numbers at the top right corner of (E) to (L) denote the pattern correlations of DPLE or DPLE_NoVolc with the observations over the tropical Pacific [20°S to 20°N, 120°E to 80°W; the region is denoted by the black box in (L)]. The stippling indicates insignificant ensemble mean anomalies at the 90% confidence level based on bootstrapping across ensemble members (see Materials and Methods).

Pacific, the pattern correlations between predicted and observed SST anomalies over the entire tropical Pacific basin (20°S to 20°N, 120°E to 80°W) are considerably higher in DPLE_NoVolc than DPLE for the El Chichón and Pinatubo eruptions (Fig. 3, E to L). Looking beyond the tropical Pacific, the observed negative SAT anomalies over South Asia and tropical Africa during these three pentads appear to be related to volcanic forcing and are better predicted in DPLE than DPLE_NoVolc, consistent with the ACC results shown in Fig. 1. However, the observed warming over the Amazon basin during 1961–1965 and 1991–1995 might be related to the El Niño–like SST warming and is thus better captured by DPLE_NoVolc.

Mechanisms of volcanic effect on tropical Pacific predictions

To explore the processes by which strong volcanic eruptions cause negative pentadal SST anomalies in the central-eastern tropical Pacific in DPLE relative to DPLE_NoVolc, we examine the monthly detrended SST evolution using a mixed-layer ocean heat budget to understand the ocean temperature tendencies (see Materials and Methods for analysis details). Results for Pinatubo are shown in Fig. 4, and those for Agung and El Chichón are shown in figs. S10 and S11. In observations, the SST in this region is characterized by a sequence of positive anomalies during 1991–1995 that are highly correlated with the Niño 3.4 index (cf. Fig. 4A and fig. S12A), suggesting a strong association with El Niño events. The ensemble mean differences between DPLE and DPLE_NoVolc from 10 different start dates ranging from 1 November 1985 to 1 November 1994 typically show three stages of SST change (Fig. 4A) in response to the volcanic forcing (Fig. 4B). These include (i) a weak negative tendency during June 1991 to December 1991, (ii) a weak positive tendency during January 1992 to December 1992, and (iii) a negative tendency during January 1993 to January 1995. The resultant ~2-year tropical Pacific cooling response during 1993–mid-1995 is key to understanding the 5- or 10-year average differences between DPLE and DPLE_NoVolc. The cooling response during 1993–mid-1995 tends to be stronger in the forecasts with start dates before the volcanic eruption than those initialized during the volcanic eruptions, presumably because of the joint effects of the full duration of volcanic forcing and initial condition memory loss.

Figure 4 (C to E) shows the ocean mixed-layer heat budget analysis composited for the six forecast ensembles initialized before the Pinatubo eruption (1 November 1985 through 1 November 1990) in the DPLE, DPLE_NoVolc, and their difference (see Materials and Methods). The initially stronger central-eastern tropical Pacific SST cooling in the DPLE relative to DPLE_NoVolc is driven by the surface shortwave reduction related to the volcanic forcing (Fig. 4C). The second phase of relative SST warming (Fig. 4D) is driven by the dynamical terms ($-u'\frac{\partial \overline{T}}{\partial x}$, $-\overline{v}\frac{\partial T'}{\partial y}$, and $-\overline{w}\frac{\partial T'}{\partial z}$), indicative of the development of El Niño, but the warming is damped by thermodynamic processes (shortwave, longwave, and latent heat fluxes). The third phase of relative SST cooling (Fig. 4E) is driven by dynamical processes, suggestive of a multiyear La Niña response to the second warming phase. The dynamically driven cooling is damped by the latent heat flux, reflecting the negative evaporation–SST feedback. The heat budget results for the first two stages are consistent with previous findings based on CESM1 experiments (*37*), but we extend it to years 2 to 3 after an eruption. The multiyear La Niña–related cooling following a weak El Niño–related warming deviates from the typical ENSO cycle simulated by CESM1 in which multiyear La Niña tends to follow strong El Niño (*38*, *39*). This is because the shortwave reduction associated with the volcanic forcing weakens the El Niño SST warming but strengthens the subsequent La Niña SST cooling, in contrast to the negative shortwave radiation–SST feedback that damps SST anomalies in a typical ENSO cycle (*37*, *40*). The trigger of initial El Niño SST warming is linked to the development of westerly wind anomalies over the western equatorial Pacific during the summer of 1991, which deepens the thermocline depth in the eastern equatorial Pacific (fig. S13E). These wind anomalies appear to be related to a rapid SST cooling response over the Maritime Continent and equatorial Indian and Atlantic Oceans after the beginning of the Pinatubo eruption (fig. S13E). The origin of these westerly anomalies has also been attributed to the stronger cooling response over land (e.g., Maritime Continent or Africa) than ocean in previous modeling studies (*41*–*43*).

The volcanic forcing induces a very similar response in forecasts initialized near the El Chichón eruption as in the Pinatubo case (fig. S11), while the response to Agung is weak and less consistent across forecast ensembles with different initialization dates (fig. S10), possibly because of factors such as the seasonal timing of eruption (*37*). We also tested the sensitivity of heat budget results to the choice of region. The Niño 3.4 region shows overall similar results as the tropical central-eastern Pacific, but the first-stage cooling is very weak because the ocean warming driven by dynamical terms counteracts the cooling induced by the shortwave reduction (fig. S12). The central tropical Pacific (20°S to 20°N, 160°E to 140°W), where the skill degradation is strongest (Fig. 1, G to I), is more affected by the shortwave process than the central-eastern tropical Pacific and shows a diminished El Niño–driven warming in the second stage (fig. S14). These analyses show that strong volcanic forcing induces tropical central-eastern Pacific cooling via a robust sequence of thermodynamic and dynamic processes in the model. The discrepancy with observed ENSO variability following these eruptions suggests that the CESM1 response to volcanic forcing overwhelms the internal ENSO variability in the tropical Pacific.

DISCUSSION

Issues in attributing observed tropical Pacific SST variability to volcanic eruptions

The degradation of the multiyear-to-decadal prediction skill of tropical Pacific SSTs by volcanic forcing in CESM1 raises general questions about the model's ability to realistically simulate the volcanic effect on ENSO and tropical Pacific decadal variability. Here, we find that CESM1 tends to produce a consistent response to historical volcanic eruptions consisting of an initially weak tropical Pacific cooling phase in the first 6 months after the eruption, followed by a 1-year weak El Niño–like warming and a subsequent multiyear La Niña–like cooling response. In some cases, the simulated volcanic response happens to amplify the observed ENSO amplitude [e.g., observed strong El Niño and subsequent 2-year La Niña during 1982–1985 (fig. S15, A and E)], while in other cases, it does not [e.g., observed El Niño in 1993–1994 (fig. S13, A and E) and observed El Niño and subsequent La Niña in 1963–1964 (fig. S16A and E)]. The effect of strong volcanic eruptions on ENSO has been widely studied using observational data,

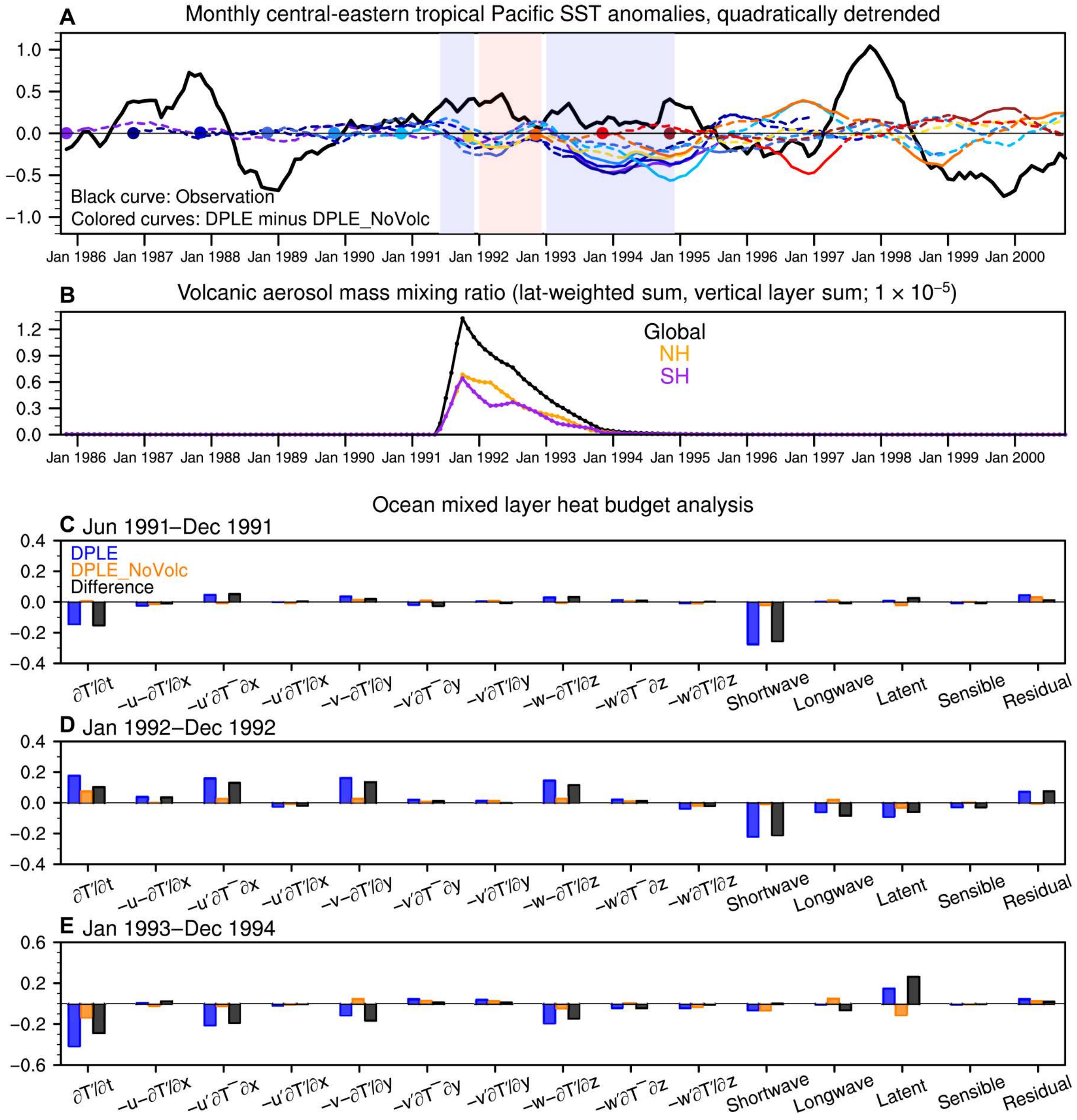

Fig. 4. Understanding how the Pinatubo eruption causes multiyear-to-decadal tropical Pacific sea surface temperature (SST) cooling in CESM1. (**A**) Time series of monthly detrended SST (°C) anomalies over the central-eastern tropical Pacific (20°S to 20°N, 160°E to 80°W) in observations (thick black curve) and the ensemble mean difference between Decadal Prediction Large Ensemble (DPLE) and DPLE_NoVolc (colored curves) in the forecasts from 10 initial dates [1 November 1985 to 1 November 1994 denoted by colored dots; the solid portion of the curves indicates differences that are significant at the 90% confidence level based on bootstrapping across ensemble members (see Materials and Methods)]. The light blue and red shaded blocks denote the phases of negative and positive SST tendencies, respectively. (**B**) Monthly time series of volcanic aerosol mass mixing ratios for the globe (black curve), the Northern Hemisphere (NH; orange curve), and the Southern Hemisphere (SH; purple curve). (**C** to **E**) Ensemble mean ocean mixed-layer heat balance terms (see Materials and Methods) averaged in the same region and integrated over three stages (June 1991 to December 1991, January 1992 to December 1992, and January 1993 to December 1994; °C) composited for the six ensembles initialized in November 1985 to November 1990 in DPLE (blue), DPLE_NoVolc (orange), and their difference (black).

paleoclimate proxies, and climate models [see a recent review by McGregor *et al.* (*44*) and references therein). It is hard to extract a statistically significant ENSO response to volcanic forcing from short observational records, especially because several volcanic eruptions (e.g., El Chichón and Pinatubo) occurred after the initiation of El Niño events (*44*). The most recent paleoclimate data reconstructions suggest that there is no consistent ENSO response to volcanic forcing (*45*, *46*). Multimodel analysis suggests that there is a robust increase in the probability of El Niño development in the year of the volcanic eruption (*43*), but the strength of this evidence depends on the fidelity of the models and the volcanic forcing (*20*, *23*).

It has been suggested that volcanic forcing can modulate Pacific decadal variability and associated impacts on GMST (*47*, *48*).

However, proxy model or observation model comparisons (*4*, *21*) suggest that the GMST cooling response is overestimated by some models even when considering the sampling issues because of El Niño phases (*16*). The CESM1 simulates tropical Pacific decadal cooling in the forecasts initialized in the 1980s and 1990s in response to volcanic forcing, while the observations show decadal warming in these periods (Fig. 2C). Thus, both previous studies and ours indicate that model fidelity needs to be carefully considered when attributing observed Pacific interannual and decadal variability to volcanic eruptions.

Potential to improve tropical Pacific multiyear-to-decadal prediction skill

Decadal prediction systems have long-standing issues in predicting Pacific SST variations (*31*–*33*, *49*, *50*). Here, we show that the CESM1 decadal prediction system without volcanic forcing exhibits a high prediction skill for tropical Pacific multiyear-to-decadal climate variability. Future research is required to determine whether other CMIP5/6 models might also show a high decadal prediction skill in the tropical Pacific that is obscured by volcanic forcing. Although a full skill assessment (e.g., ACC scores) requires repeating computationally expensive hindcasts without volcanic forcing for the past several decades, it could be useful to check whether selected forecasts initialized before the large historical volcanic eruptions (*26*, *28*) show poor performance in predicting observed tropical Pacific decadal SST anomalies, similar to analysis in Fig. 3. The high skill of the CESM1 no-volcano forecasts suggests that there is latent predictability associated with internal tropical decadal variability. The mechanisms giving rise to this predictability remain unclear, but they are likely related to slow oceanic processes (*25*, *51*, *52*). Tropical Pacific decadal SST variations can affect surface temperature, hydroclimate, and marine ecosystems across the globe via atmospheric and oceanic teleconnections, and therefore, improved understanding of prediction system behavior in the tropical Pacific is crucial for advancing Earth system prediction on decadal time scales.

MATERIALS AND METHODS

Initialized forecasts and uninitialized simulations

To explore the influence of volcanic forcing on multiyear-to-decadal predictions, we compare the CESM1 DPLE (*31*) with a parallel set of decadal forecasts that exclude historical volcanic forcing (DPLE_NoVolc). All forecasts use the same model version and configuration used for the CESM1 LE (*34*) at nominal 1° horizontal resolutions. The DPLE consists of 40-member retrospective forecasts initialized on 1 November each year during 1954–2015 and integrated for 122 months. Ensemble forecasts are initialized from identical ocean and sea ice conditions, and ensemble spread is created by adding round-off level perturbations to the atmospheric temperature initial conditions. The ocean and sea ice initial conditions are generated by forcing the ocean and sea ice model components of CESM1 with historical atmospheric and surface flux fields, while the atmosphere and land initial conditions are obtained from the CESM1 LE. All forecasts are run using CMIP5 "historical" forcings for 1954–2005 and CMIP5 representative concentration pathway 8.5 forcing thereafter. DPLE_NoVolc follows the DPLE protocol except that it excludes volcanic aerosol forcing during 1954–2005, and it has a smaller ensemble size of 10. Note that DPLE_NoVolc uses the same historical initial conditions as in DPLE, and in particular, the ocean initial conditions include the effects of volcanic forcing. Both systems should have identical initial condition–related predictability (that includes volcanic influence), and the difference between DPLE_NoVolc and DPLE should reflect differences in how volcanic aerosol forcing interacts with the model state during forward integration. The volcanic aerosol forcing data used in DPLE include the three major tropical volcanic eruptions (Mt. Agung in March 1963, El Chichón in April 1982, and Mt. Pinatubo in June 1991) and two small eruptions in the 1970s, as shown by the time series of globally and vertically integrated volcanic aerosol mass mixing ratio (Fig. 2D). The time and latitudinally varying volcanic forcing is based on Ammann *et al.* (*53*). We also compare the DPLE with the uninitialized CESM1 LE to isolate the role of initialization in affecting the prediction skill (*31*). The CESM1 LE is composed of 40-member historical simulations subject to CMIP5 forcing during 1920–2100, including the historical volcanic forcing. We examine CESM1 LE results from the period of 1954–2015 to be consistent with the initialized forecasts.

Drift correlation and detrending methods

The drifting climatology of the ensemble forecasts is calculated by averaging the ensemble mean forecasts across 1964–2015 for each lead time, for either monthly means [forecast months 1 to 122 (FM1–122)] or annual means (FY1–10). The period 1964–2015 was used as the baseline climatology because it is the longest temporal window for which the same-size (52 years) sample exists for each lead time. Drift-corrected anomalies are obtained by removing the lead time–dependent climatology from each ensemble member. The anthropogenic climate change is estimated by a quadratic fit (*21*) of the ensemble mean forecast anomalies across 1954–2015 as a function of lead time (i.e., FY1–10 or FM1–122) and is removed from individual drift-corrected ensemble members to obtain detrended anomalies. For observations and LE, the climatology and trend are calculated over the same time periods as for the forecasts. We also adopted two other methods to estimate anthropogenic climate change based on the lead time–dependent linear trend (*31*) and the ensemble mean of the uninitialized CESM1 LE (*33*). However, removing the ensemble mean of LE also removes the effect of volcanic forcing in addition to anthropogenic climate change, and so, this is not a preferred method in the context of the present analysis.

Observational datasets and forecast verification

The hindcasts are verified against the National Oceanic and Atmospheric Administration (NOAA) Extended Reconstruction Sea Surface Temperature version 5 (ERSSTv5) dataset at 2° spatial resolution (*54*) for SST and the Berkeley Earth Surface Temperature dataset at 1° spatial resolution (*55*) for near SAT. The observational data are regridded to the model grid before verification. The prediction skill is assessed using the ACC and root mean square error (RMSE) between the ensemble mean forecasts and observations. The significance of ACC or ensemble mean anomalies is tested using the nonparametric bootstrap method. Following Goddard *et al.* (*50*) and Yeager *et al.* (*31*), we tested whether an ACC or ACC difference is significantly different from zero using a bootstrapped distribution of 5000 values at each spatial location or for area average indices by resampling (with replacement) across both the time and the ensemble member dimensions. Similarly, the

significance of ensemble mean anomalies at a given time is tested by resampling (with replacement) across the ensemble member dimension. The 5000 values are calculated on the basis of 10-member ensembles for all model analyses to account for the different ensemble sizes among DPLE (40 ensemble members), DPLE_NoVolc (10 ensemble members), and LE (40 ensemble members). A positive value is significant at the 90% confidence level if its bootstrapped distribution has fewer than 500 values that are below zero ($P < 500/5000 = 0.1$), and vice versa for a negative value. We apply Fisher's z transformations to ACC scores before calculating P values.

Signal-to-noise paradox

The predictability (quantified by the signal-to-total variance ratio) has been found to be higher in observation than in climate models, which is known as the signal-to-noise paradox (*33*, *56*). We evaluate whether this is the case in DPLE and DPLE_NoVolc. The predictable variance fraction (PVF;$\text{PVF} = \sigma^2_{\text{signal}}/\sigma^2_{\text{total}}$) is defined as the relative fraction of signal (ensemble mean) variance to total variance (averaged across individual ensemble members). The estimation of the model's signal variance (σ^2_{signal}) might be biased due to a finite ensemble, especially for the 10-member DPLE_NoVolc. SD of total variability (σ_{total}) is the square root of total variance (σ^2_{total}). The RPC ($\text{RPC} = \text{ACC}/\sqrt{\text{PVF}}$) quantifies the mismatch between the potentially predictable component determined from the model ensemble spread and the predictable component of observed variability as quantified by ACC. We do not calculate RPC where ACC is negative, following Smith *et al.* (*33*), but note that RPC is not meaningful when the ACC is positive but insignificant (*57*). RPC is expected to be underestimated using a finite ensemble, because of the combined effects of underestimation of ACC and overestimation of σ^2_{signal} due to an insufficient noise suppression (*56*, *57*). A 40-member DPLE ensemble should be sufficient to estimate an accurate RPC, but a 10-member DPLE_NoVolc ensemble might not be (*57*). For a sufficiently large ensemble, RPC should be 1 when the correlation skill matches the square root of the predictable portion of variance in the model. RPC less than 1 indicates that the actual prediction skill is lower than what would be expected from the model's PVF, while RPC greater than 1 implies that the signal-to-noise ratio is underestimated in the model compared to observations.

Ocean mixed-layer heat budget analysis

To decompose the processes by which the volcanic forcing affects the tropical Pacific SST anomalies in forecasts, we conducted an ocean mixed-layer heat budget analysis for the monthly detrended ensemble mean anomalies in the DPLE, DPLE_NoVolc, and their difference. We calculate the heat budget at each horizontal grid point as an approximate balance between the monthly mean heat storage tendency, ocean temperature advection, and surface heat fluxes according to the following equation

$$\frac{\partial\langle T'\rangle}{\partial t} = \left\langle -\overline{u}\frac{\partial T'}{\partial x}\right\rangle + \left\langle -u'\frac{\partial \overline{T}}{\partial x}\right\rangle + \left\langle -u'\frac{\partial T'}{\partial x}\right\rangle + \left\langle -\overline{v}\frac{\partial T'}{\partial y}\right\rangle + \left\langle -v'\frac{\partial \overline{T}}{\partial y}\right\rangle + \left\langle -v'\frac{\partial T'}{\partial y}\right\rangle + \left\langle -\overline{w}\frac{\partial T'}{\partial z}\right\rangle + \left\langle -w'\frac{\partial \overline{T}}{\partial z}\right\rangle + \left\langle -w'\frac{\partial T'}{\partial z}\right\rangle + \frac{Q'_{\text{net}}}{\rho C_p H} + R$$

$$\langle * \rangle = \frac{1}{H}\int_{-H}^{0} * \, dz$$

where T is the mixed-layer temperature; u, v, and w are the resolved ocean currents in the zonal (x), meridional (y), and vertical (z) directions, respectively; Q'_{net} is the net surface heat flux (sum of shortwave, longwave, latent, and sensible heat fluxes); ρ is the ocean water density (1030 kg m^{-3}); C_p is the ocean heat capacity (4000 J kg^{-1} K^{-1}); H is the climatological mixed-layer depth that is averaged across FM1 to FM122 but varies with latitude and longitude; R represents the residual term, including diffusion and subgrid scale terms; the overbar denotes the monthly drifting climatology; and the prime denotes the monthly drift-corrected and detrended anomaly.

Supplementary Materials

This PDF file includes:
Supplementary Text
Figs. S1 to S16

REFERENCES AND NOTES

1. A. Robock, Volcanic eruptions and climate. *Rev. Geophys.* **38**, 191–219 (2000).
2. C. Timmreck, Modeling the climatic effects of large explosive volcanic eruptions. *Wiley Interdiscip. Rev. Clim. Chang.* **3**, 545–564 (2012).
3. D. T. Shindell, G. A. Schmidt, M. E. Mann, G. Faluvegi, Dynamic winter climate response to large tropical volcanic eruptions since 1600. *J. Geophys. Res.* **109**, D05104 (2004).
4. K. J. Anchukaitis, B. M. Buckley, E. R. Cook, B. I. Cook, R. D. D'Arrigo, C. M. Ammann, Influence of volcanic eruptions on the climate of the Asian monsoon region. *Geophys. Res. Lett.* **37**, L22703 (2010).
5. O. H. Otterå, M. Bentsen, H. Drange, L. Suo, External forcing as a metronome for Atlantic multidecadal variability. *Nat. Geosci.* **3**, 688–694 (2010).
6. W. Man, M. Zuo, T. Zhou, J. T. Fasullo, I. Bethke, X. Chen, L. Zou, B. Wu, Potential influences of volcanic eruptions on future global land monsoon precipitation changes. *Earth's Future* **9**, (2021).
7. J. A. Church, N. J. White, J. M. Arblaster, Significant decadal-scale impact of volcanic eruptions on sea level and ocean heat content. *Nature* **438**, 74–77 (2005).
8. G. Stenchikov, T. L. Delworth, V. Ramaswamy, R. J. Stouffer, A. Wittenberg, F. Zeng, Volcanic signals in oceans. *J. Geophys. Res.* **114**, D16104 (2009).
9. Y. Ding, J. A. Carton, G. A. Chepurin, G. Stenchikov, A. Robock, L. T. Sentman, J. P. Krasting, Ocean response to volcanic eruptions in Coupled Model Intercomparison Project 5 simulations. *J. Geophys. Res. Oceans* **119**, 5622–5637 (2014).
10. J. T. Fasullo, R. Tomas, S. Stevenson, B. Otto-Bliesner, E. Brady, E. Wahl, The amplifying influence of increased ocean stratification on a future year without a summer. *Nat. Commun.* **8**, 1236 (2017).
11. D. Zanchettin, O. Bothe, H. F. Graf, S. J. Lorenz, J. Luterbacher, C. Timmreck, J. H. Jungclaus, Background conditions influence the decadal climate response to strong volcanic eruptions. *J. Geophys. Res. Atmos.* **118**, 4090–4106 (2013).
12. A. Robock, J. Mao, The volcanic signal in surface temperature observations. *J. Clim.* **8**, 1086–1103 (1995).
13. B. D. Santer, T. M. L. Wigley, C. Doutriaux, J. S. Boyle, J. E. Hansen, P. D. Jones, G. A. Meehl, E. Roeckner, S. Sengupta, K. E. Taylor, Accounting for the effects of volcanoes and ENSO in comparisons of modeled and observed temperature trends. *J. Geophys. Res. Atmos.* **106**, 28033–28059 (2001).

14. B. D. Santer, S. Solomon, C. Bonfils, M. D. Zelinka, J. F. Painter, F. Beltran, J. C. Fyfe, G. Johannesson, C. Mears, D. A. Ridley, J. Vernier, F. J. Wentz, Observed multivariable signals of late 20th and early 21st century volcanic activity. *Geophys. Res. Lett.* **42**, 500–509 (2015).
15. D. W. J. Thompson, J. M. Wallace, P. D. Jones, J. J. Kennedy, Identifying signatures of natural climate variability in time series of global-mean surface temperature: Methodology and insights. *J. Clim.* **22**, 6120–6141 (2009).
16. F. Lehner, A. P. Schurer, G. C. Hegerl, C. Deser, T. L. Frölicher, The importance of ENSO phase during volcanic eruptions for detection and attribution. *Geophys. Res. Lett.* **43**, 2851–2858 (2016).
17. J. B. Adams, M. E. Mann, C. M. Ammann, Proxy evidence for an El Niño-like response to volcanic forcing. *Nature* **426**, 274–278 (2003).
18. J. Li, S.-P. Xie, E. R. Cook, M. S. Morales, D. A. Christie, N. C. Johnson, F. Chen, R. D'Arrigo, A. M. Fowler, X. Gou, K. Fang, El Niño modulations over the past seven centuries. *Nat. Clim. Chang.* **3**, 822–826 (2013).
19. S. Driscoll, A. Bozzo, L. J. Gray, A. Robock, G. Stenchikov, Coupled Model Intercomparison Project 5 (CMIP5) simulations of climate following volcanic eruptions. *J. Geophys. Res. Atmos.* **117**, D17105 (2012).
20. S. Stevenson, B. Otto-Bliesner, J. Fasullo, E. Brady, "El Niño like" hydroclimate responses to last millennium volcanic eruptions. *J. Clim.* **29**, 2907–2921 (2016).
21. G. Liguori, S. McGregor, J. M. Arblaster, M. S. Singh, G. A. Meehl, A joint role for forced and internally-driven variability in the decadal modulation of global warming. *Nat. Commun.* **11**, 3827 (2020).
22. D. Zanchettin, C. Timmreck, M. Khodri, A. Schmidt, M. Toohey, M. Abe, S. Bekki, J. Cole, S.-W. Fang, W. Feng, G. Hegerl, B. Johnson, N. Lebas, A. N. Le Grande, G. W. Mann, L. Marshall, L. Rieger, A. Robock, S. Rubinetti, K. Tsigaridis, H. Weierbach, Effects of forcing differences and initial conditions on inter-model agreement in the VolMIP volc-pinatubo-full experiment. *Geosci. Model Dev.* **15**, 2265–2292 (2022).
23. C. Gao, A. Robock, C. Ammann, Volcanic forcing of climate over the past 1500 years: An improved ice core-based index for climate models. *J. Geophys. Res. Atmos.* **113**, D23111 (2008).
24. D. Zanchettin, C. Timmreck, M. Toohey, J. H. Jungclaus, M. Bittner, S. J. Lorenz, A. Rubino, Clarifying the relative role of forcing uncertainties and initial-condition unknowns in spreading the climate response to volcanic eruptions. *Geophys. Res. Lett.* **46**, 1602–1611 (2019).
25. G. A. Meehl, L. Goddard, G. Boer, R. Burgman, G. Branstator, C. Cassou, S. Corti, G. Danabasoglu, F. Doblas-Reyes, E. Hawkins, A. Karspeck, M. Kimoto, A. Kumar, D. Matei, J. Mignot, R. Msadek, A. Navarra, H. Pohlmann, M. Rienecker, T. Rosati, E. Schneider, D. Smith, R. Sutton, H. Teng, G. J. van Oldenborgh, G. Vecchi, S. Yeager, Decadal climate prediction: An update from the trenches. *Bull. Am. Meteorol. Soc.* **95**, 243–267 (2014).
26. G. J. Boer, D. M. Smith, C. Cassou, F. Doblas-Reyes, G. Danabasoglu, B. Kirtman, Y. Kushnir, M. Kimoto, G. A. Meehl, R. Msadek, W. A. Mueller, K. E. Taylor, F. Zwiers, M. Rixen, Y. Ruprich-Robert, R. Eade, The Decadal Climate Prediction Project (DCPP) contribution to CMIP6. *Geosci. Model Dev.* **9**, 3751–3777 (2016).
27. I. Bethke, S. Outten, O. H. Otterå, E. Hawkins, S. Wagner, M. Sigl, P. Thorne, Potential volcanic impacts on future climate variability. *Nat. Clim. Chang.* **7**, 799–805 (2017).
28. L. Hermanson, R. Bilbao, N. Dunstone, M. Ménégoz, P. Ortega, H. Pohlmann, J. I. Robson, D. M. Smith, G. Strand, C. Timmreck, S. Yeager, G. Danabasoglu, Robust multiyear climate impacts of volcanic eruptions in decadal prediction systems. *J. Geophys. Res. Atmos.* **125**, e2019JD031739 (2020).
29. G. A. Meehl, H. Teng, N. Maher, M. H. England, Effects of the Mount Pinatubo eruption on decadal climate prediction skill of Pacific sea surface temperatures. *Geophys. Res. Lett.* **42**, 10840–10846 (2015).
30. S. Illing, C. Kadow, H. Pohlmann, C. Timmreck, Assessing the impact of a future volcanic eruption on decadal predictions. *Earth Syst. Dynam.* **9**, 701–715 (2018).
31. S. G. Yeager, G. Danabasoglu, N. A. Rosenbloom, W. Strand, S. C. Bates, G. A. Meehl, A. R. Karspeck, K. Lindsay, M. C. Long, H. Teng, N. S. Lovenduski, Predicting near-term changes in the Earth System: A large ensemble of initialized decadal prediction simulations using the Community Earth System Model. *Bull. Am. Meteorol. Soc.* **99**, 1867–1886 (2018).
32. B. Kirtman, S. Power, A. Adedoyin, G. Boer, R. Bojariu, Near-term climate change: Projections and predictability, in *Climate Change 2013: The Physical Science Basis*, T. F. Stocker, D. Qin, G.-K. Plattner, M. Tignor, S. K. Allen, J. Boschung, A. Nauels, Y. Xia, V. Bex, P. M. Midgley, Eds. (Cambridge Univ. Press, 2013), pp. 953–1028.
33. D. M. Smith, R. Eade, A. A. Scaife, L.-P. Caron, G. Danabasoglu, T. M. Del Sole, T. Delworth, F. J. Doblas-Reyes, N. J. Dunstone, L. Hermanson, V. Kharin, M. Kimoto, W. J. Merryfield, T. Mochizuki, W. A. Müller, H. Pohlmann, S. Yeager, X. Yang, Robust skill of decadal climate predictions. *NPJ Clim. Atmos. Sci.* **2**, 13 (2019).
34. J. E. Kay, C. Deser, A. Phillips, A. Mai, C. Hannay, G. Strand, J. M. Arblaster, S. C. Bates, G. Danabasoglu, J. Edwards, M. Holland, P. Kushner, J.-F. Lamarque, D. Lawrence, K. Lindsay, A. Middleton, E. Munoz, R. Neale, K. Oleson, L. Polvani, M. Vertenstein, The Community Earth System Model (CESM) Large Ensemble Project: A community resource for studying climate change in the presence of internal climate variability. *Bull. Am. Meteorol. Soc.* **96**, 1333–1349 (2015).
35. A. Capotondi, C. Deser, A. Phillips, Y. Okumura, S. Larson, ENSO and Pacific decadal variability in the Community Earth System Model Version 2. *J. Adv. Model. Earth Syst.* **12**, e2019MS002022 (2020).
36. X. Wu, Y. M. Okumura, P. N. DiNezio, S. G. Yeager, C. Deser, The equatorial Pacific cold tongue bias in CESM1 and its influence on ENSO forecasts. *J. Clim.* **35**, 3261–3277 (2022).
37. S. Stevenson, J. T. Fasullo, B. L. Otto-Bliesner, R. A. Tomas, C. Gao, Role of eruption season in reconciling model and proxy responses to tropical volcanism. *Proc. Natl. Acad. Sci. U.S.A.* **114**, 1822–1826 (2017).
38. P. N. DiNezio, C. Deser, Y. Okumura, A. Karspeck, Predictability of 2-year La Niña events in a coupled general circulation model. *Clim. Dyn.* **49**, 4237–4261 (2017).
39. X. Wu, Y. M. Okumura, P. N. DiNezio, What controls the duration of El Niño and La Niña events? *J. Clim.* **32**, 5941–5965 (2019).
40. N. Maher, S. McGregor, M. H. England, A. S. Gupta, Effects of volcanism on tropical variability. *Geophys. Res. Lett.* **42**, 6024–6033 (2015).
41. M. Ohba, H. Shiogama, T. Yokohata, M. Watanabe, Impact of strong tropical volcanic eruptions on ENSO simulated in a coupled GCM. *J. Clim.* **26**, 5169–5182 (2013).
42. E. Predybaylo, G. L. Stenchikov, A. T. Wittenberg, F. Zeng, Impacts of a Pinatubo-size volcanic eruption on ENSO. *J. Geophys. Res. Atmos.* **122**, 925–947 (2017).
43. M. Khodri, T. Izumo, J. Vialard, S. Janicot, C. Cassou, M. Lengaigne, J. Mignot, G. Gastineau, E. Guilyardi, N. Lebas, A. Robock, M. J. McPhaden, Tropical explosive volcanic eruptions can trigger El Niño by cooling tropical Africa. *Nat. Commun.* **8**, 778 (2017).
44. S. McGregor, M. Khodri, N. Maher, M. Ohba, F. S. R. Pausata, S. Stevenson, The effect of strong volcanic eruptions on ENSO, in *El Niño Southern Oscillation in a Changing Climate*, M. J. McPhaden, A. Santoso, W. Cai, Eds. (Wiley, 2020), pp. 267–287.
45. S. G. Dee, K. M. Cobb, J. Emile-Geay, T. R. Ault, R. L. Edwards, H. Cheng, C. D. Charles, No consistent ENSO response to volcanic forcing over the last millennium. *Science* **367**, 1477–1481 (2020).
46. F. Zhu, J. Emile-Geay, K. J. Anchukaitis, G. J. Hakim, A. T. Wittenberg, M. S. Morales, M. Toohey, J. King, A re-appraisal of the ENSO response to volcanism with paleoclimate data assimilation. *Nat. Commun.* **13**, 747 (2022).
47. D. M. Smith, B. B. B. Booth, N. J. Dunstone, R. Eade, L. Hermanson, G. S. Jones, A. A. Scaife, K. L. Sheen, V. Thompson, Role of volcanic and anthropogenic aerosols in the recent global surface warming slowdown. *Nat. Clim. Chang.* **6**, 936–940 (2016).
48. L. Dong, T. Zhou, X. Chen, Changes of Pacific decadal variability in the twentieth century driven by internal variability, greenhouse gases, and aerosols. *Geophys. Res. Lett.* **41**, 8570–8577 (2014).
49. F. J. Doblas-Reyes, I. Andreu-Burillo, Y. Chikamoto, J. García-Serrano, V. Guemas, M. Kimoto, T. Mochizuki, L. R. L. Rodrigues, G. J. van Oldenborgh, Initialized near-term regional climate change prediction. *Nat. Commun.* **4**, 1715 (2013).
50. L. Goddard, A. Kumar, A. Solomon, D. Smith, G. Boer, P. Gonzalez, V. Kharin, W. Merryfield, C. Deser, S. J. Mason, B. P. Kirtman, R. Msadek, R. Sutton, E. Hawkins, T. Fricker, G. Hegerl, C. A. T. Ferro, D. B. Stephenson, G. A. Meehl, T. Stockdale, R. Burgman, A. M. Greene, Y. Kushnir, M. Newman, J. Carton, I. Fukumori, T. Delworth, A verification framework for interannual-to-decadal predictions experiments. *Clim. Dyn.* **40**, 245–272 (2013).
51. Z. Liu, E. D. Lorenzo, Mechanisms and predictability of Pacific decadal variability. *Curr. Clim. Change Rep.* **4**, 128–144 (2018).
52. S. Power, M. Lengaigne, A. Capotondi, M. Khodri, J. Vialard, B. Jebri, E. Guilyardi, S. McGregor, J.-S. Kug, M. Newman, M. J. McPhaden, G. Meehl, D. Smith, J. Cole, J. Emile-Geay, D. Vimont, A. T. Wittenberg, M. Collins, G.-I. Kim, W. Cai, Y. Okumura, C. Chung, K. M. Cobb, F. Delage, Y. Y. Planton, A. Levine, F. Zhu, J. Sprintall, E. D. Lorenzo, X. Zhang, J.-J. Luo, X. Lin, M. Balmaseda, G. Wang, B. J. Henley, Decadal climate variability in the tropical Pacific: Characteristics, causes, predictability, and prospects. *Science* **374**, eaay9165 (2021).
53. C. M. Ammann, G. A. Meehl, W. M. Washington, C. S. Zender, A monthly and latitudinally varying volcanic forcing dataset in simulations of 20th century climate. *Geophys. Res. Lett.* **30**, 1657 (2003).
54. B. Huang, P. W. Thorne, V. F. Banzon, T. Boyer, G. Chepurin, J. H. Lawrimore, M. J. Menne, T. M. Smith, R. S. Vose, H.-M. Zhang, Extended Reconstructed Sea Surface Temperature, Version 5 (ERSSTv5): Upgrades, validations, and intercomparisons. *J. Clim.* **30**, 8179–8205 (2017).
55. R. Rohde, R. Muller, R. Jacobsen, S. Perlmutter, A. Rosenfeld, J. Wurtele, J. Curry, C. Wickham, S. Mosher, Berkeley Earth temperature averaging process. *Geoinfor. Geostat.: An overview* **1**, 1–13 (2013).
56. A. A. Scaife, D. Smith, A signal-to-noise paradox in climate science. *NPJ Clim. Atmos. Sci.* **1**, 28 (2018).
57. K. Strommen, T. N. Palmer, Signal and noise in regime systems: A hypothesis on the predictability of the North Atlantic Oscillation. *Quart. J. R. Met. Soc.* **145**, 147–163 (2019).

Acknowledgments: We thank N. Maher and the members of NCAR's Climate Analysis Section for discussions on the analysis. **Funding:** The CESM project is supported primarily by the National Science Foundation (NSF). The National Center for Atmospheric Research (NCAR) is a major facility sponsored by the NSF under Cooperative Agreement 1852977. Portions of this study were supported by the Regional and Global Model Analysis (RGMA) component of the Earth and Environmental System Modeling Program of the U.S. Department of Energy's Office of Biological and Environmental Research (BER) via NSF IA 1844590 and under award number DE-SC0022070 and by the Department of Commerce through the Climate Variability and Predictability program of NOAA OAR's Climate Program Office under award NA20OAR4310408. This research used resources of the National Energy Research Scientific Computing Center (NERSC), a U.S. Department of Energy Office of Science User Facility located at Lawrence Berkeley National Laboratory, operated under contract no. DE-AC02-05CH11231. X.W. is supported by an Advanced Study Program postdoctoral fellowship from NCAR. **Author contributions:** X.W. and S.G.Y. conceived the study. X.W. conducted the analysis, and S.G.Y. and C.D. contributed to the interpretation of the results. S.G.Y., N.R., and G.A.M. led the design and execution of the DPLE and DPLE_NoVolc experiments. X.W. wrote the manuscript with input from all the authors. **Competing interests:** The authors declare that they have no competing interests. **Data and materials availability:** The CESM1 DPLE and LE data are available through NCAR's Climate Data Gateway at www.earthsystemgrid.org/dataset/ucar.cgd.ccsm4.CESM1-CAM5-DP.html and www.earthsystemgrid.org/dataset/ucar.cgd.ccsm4.cesmLE.html, respectively. DPLE_NoVolc data are available through https://portal.nersc.gov/archive/home/c/ccsm/www/CESM1-CAM5-DP-NoV. ERSSTv5 SSTs are available at https://psl.noaa.gov/data/gridded/data.noaa.ersst.v5.html. Berkeley Earth near-surface air temperatures are available at http://berkeleyearth.org/data/. All data needed to evaluate the conclusions in the paper are present in the paper and/or the Supplementary Materials.

Submitted 18 July 2022
Accepted 15 March 2023
Published 12 April 2023
10.1126/sciadv.add9364
