# Supplementary material for: Volcanic forcing degrades multiyear-to-decadal prediction skill in the tropical Pacific
Source: Sci Adv. 2023 Apr 12;9(15):eadd9364. doi: 10.1126/sciadv.add9364 (PMC10096591; doi:10.1126/sciadv.add9364)
Supplement: Supplementary file 2 — Supplementary Text Figs. S1 to S16 [file sciadv.add9364_sm.pdf]

Science Advances
AAAS

Supplementary Materials for

Volcanic forcing degrades multiyear-to-decadal prediction skill in the tropical Pacific

Xian Wu *et al.*

Corresponding author: Xian Wu, xianwu@ucar.edu

Sci. Adv. **9**, eadd9364 (2023)
DOI: 10.1126/sciadv.add9364

This PDF file includes:

Supplementary Text
Figs. S1 to S16

Volcanic effect on long-term drifting climatology and trend of decadal predictions

To understand the volcanic effect on long-term drifting climatology, trend, and de-drifted (and detrended) anomalies in the decadal prediction system, we compare the original SSTs (see Materials and Methods; Fig. S8A-C), de-drifted SST anomalies (Fig. S8D-F), and de-drifted and detrended SST anomalies in the central-eastern tropical Pacific (Fig. 2A-C). The original tropical Pacific SSTs are significantly lower in DPLE than DPLE_NoVolc forecasts that are initialized around the three major volcanic eruptions for FY1–5, 6–10, and 1–10. The cooling effect on these particular forecast ensembles has a cumulative effect that makes the long-term SST climatology cooler in DPLE than DPLE_NoVolc across the forecast years ranging from 1 to 10 (Fig. S9A). Due to the different lead time dependent climatologies, the de-drifted SST anomalies in forecasts starting around 1970 and 2000 become warmer in DPLE than DPLE_NoVolc, although during these periods the volcanic forcing is weak or absent (Fig. S8D-F). The drift correction process also artificially weakens the direct volcanic cooling effect around the three major volcanic eruptions. In particular, the volcano-induced cooling in the original SSTs for FY1–10 (Fig. S8C) becomes insignificant after drift correction (Fig. S8F). The drift correction process does not affect ACC but reduces RMSE by eliminating the model errors in estimating the long-term climatology. The difference in ACC between DPLE and DPLE_NoVolc is small for the non-detrended results, as the correlation is dominated by the greenhouse-induced warming trend in both observation and forecasts. After removing the quadratic trend, DPLE and DPLE_NoVolc show similar differences near the strong volcanic eruptions but the significant differences around 2010 disappear (cf., Fig. 2A-C and Fig. S8D-F). These comparisons show that volcanic forcing not only directly cools the tropical Pacific SST in the forecasts that experience the major volcanic eruptions but also

cumulatively affects the long-term climatology and trend, which can alter estimated anomalies during periods when the volcanic forcing is weak or absent.

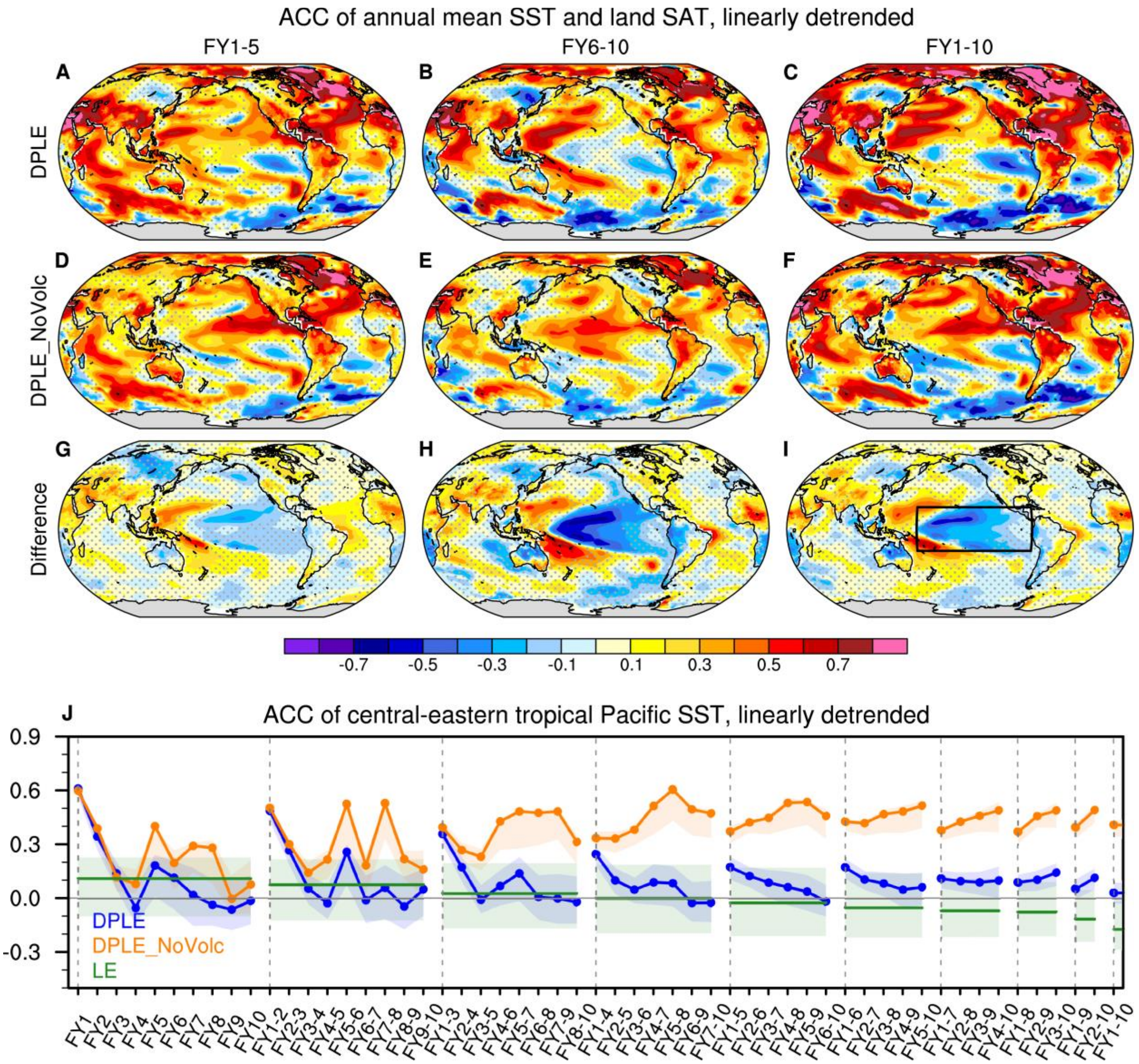

Fig. S1. Volcanic impact on the prediction skill of linearly detrended multiyear-to-decadal surface temperatures. As in Fig. 1 in the main text but for the linearly detrended results. ACC of linearly detrended annual SST and SAT over land at FY1–5, FY6–10, and FY1–10 (from left to right columns) during 1955-2015 for (**A-C**) DPLE; (**D-F**) DPLE_NoVolc (verified against the ERSSTv5 and BEST data); and (**G-I)** their difference (DPLE minus DPLE_NoVolc). The stippling indicates insignificant values at the 90% confidence level based on bootstrapping across both time and ensemble members (see Materials and Methods). (**J**) ACC of linearly detrended central-eastern tropical Pacific SST (20°S–20°N, 160°E–80°W; the region is denoted by the black box in **I**) as a function of lead time intervals corresponding to 1-yr–10-yr averages for DPLE (blue curves), DPLE_NoVolc (orange curves), and LE (green lines). Colored shading denotes the 10th–90th percentile range of ACC based on 5,000 bootstrapped ensemble means from randomly sampled 10-member ensembles, while the curves show ACC for the full ensembles (40, 10, and 40 members for DPLE, DPLE_NoVolc, and LE, respectively).

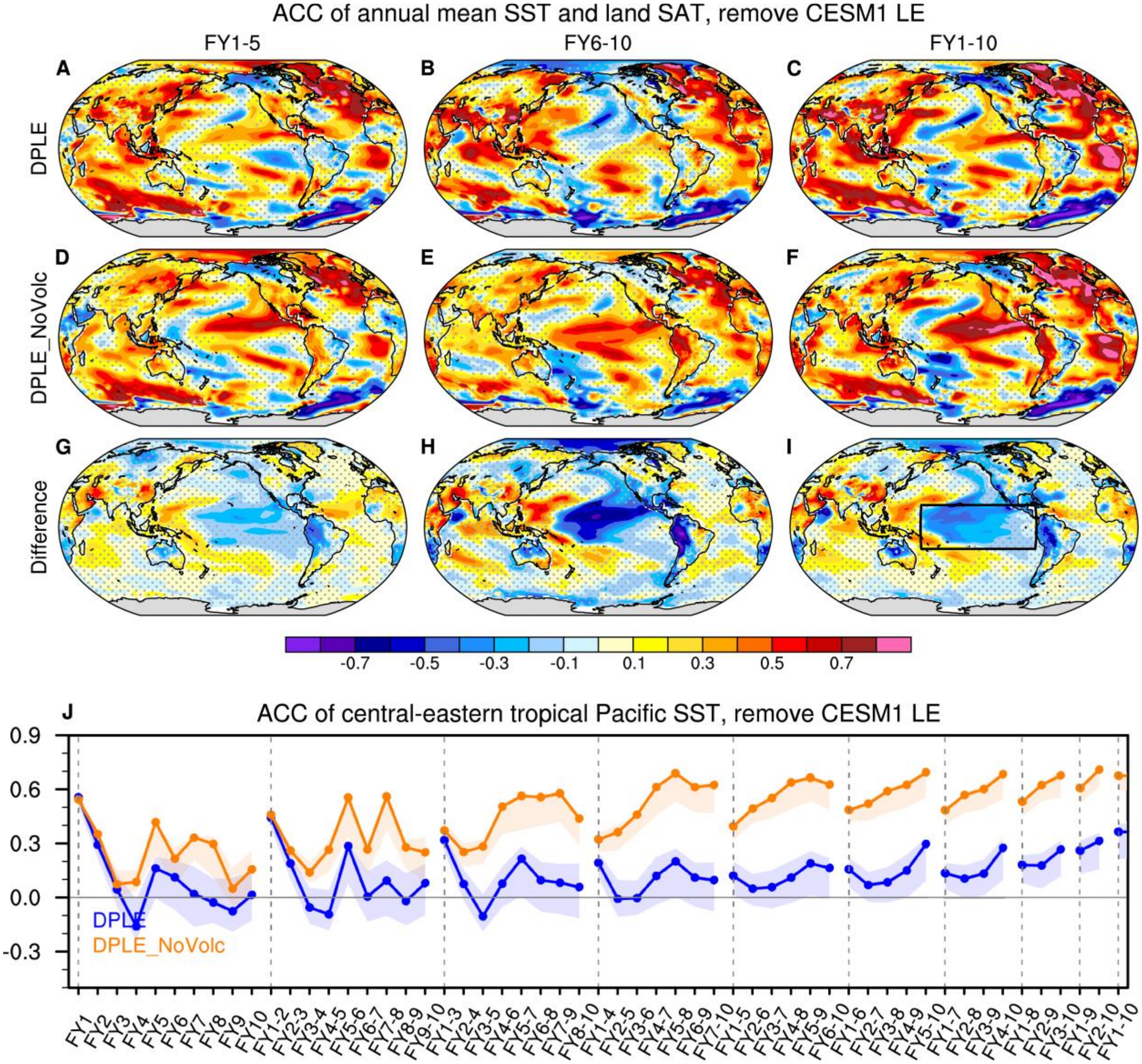

Fig. S2. Volcanic impact on the prediction skill of multiyear-to-decadal surface temperatures with the ensemble mean of LE removed. As in Fig. 1 in the main text but for the results with the ensemble mean of LE removed from DPLE, DPLE_NoVolc, and observation. ACC of annual SST and SAT over land with the ensemble mean of LE removed at FY1–5, FY6–10, and FY1–10 (from left to right columns) during 1955-2015 for (**A-C**) DPLE; (**D-F**) DPLE_NoVolc (verified against the ERSSTv5 and BEST data); and (**G-I**) their difference (DPLE minus DPLE_NoVolc). The stippling indicates insignificant values at the 90% confidence level based on bootstrapping across both time and ensemble members (see Materials and Methods). (**J**) ACC of central-eastern tropical Pacific SST (20°S–20°N, 160°E–80°W; the region is denoted by the black box in **I**) with the ensemble mean of LE removed as a function of lead time intervals corresponding to 1-yr–10-yr averages for DPLE (blue curves) and DPLE_NoVolc (orange curves). Colored shading denotes the 10th–90th percentile range of ACC based on 5,000 bootstrapped ensemble means from randomly sampled 10-member ensembles, while the curves show ACC for the full ensembles (40 and 10 members for DPLE and DPLE_NoVolc, respectively).

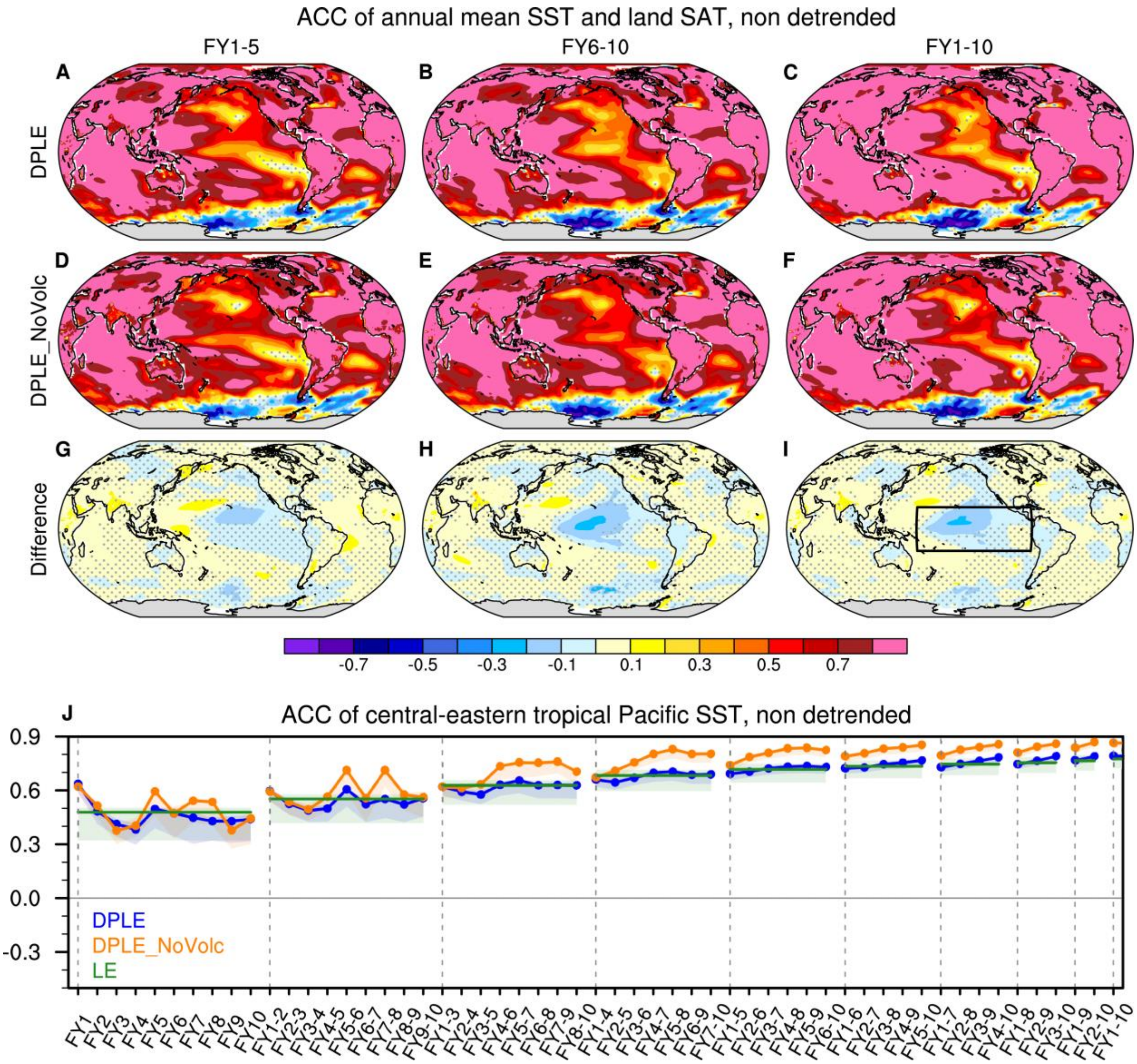

Fig. S3. Volcanic impact on the prediction skill of multiyear-to-decadal surface temperatures. As in Fig. 1 in the main text but for the non-detrended results. ACC of annual SST and SAT over land at FY1–5, FY6–10, and FY1–10 (from left to right columns) during 1955-2015 for (**A-C**) DPLE; (**D-F**) DPLE_NoVolc (verified against the ERSSTv5 and BEST data); and (**G-I)** their difference (DPLE minus DPLE_NoVolc). The stippling indicates insignificant values at the 90% confidence level based on bootstrapping across both time and ensemble members (see Materials and Methods). (**J**) ACC of central-eastern tropical Pacific SST (20°S–20°N, 160°E–80°W; the region is denoted by the black box in **I**) as a function of lead time intervals corresponding to 1-yr–10-yr averages for DPLE (blue curves), DPLE_NoVolc (orange curves), and LE (green lines). Colored shading denotes the 10th–90th percentile range of ACC based on 5,000 bootstrapped ensemble means from randomly sampled 10-member ensembles, while the curves show ACC for the full ensembles (40, 10, and 40 members for DPLE, DPLE_NoVolc, and LE, respectively).

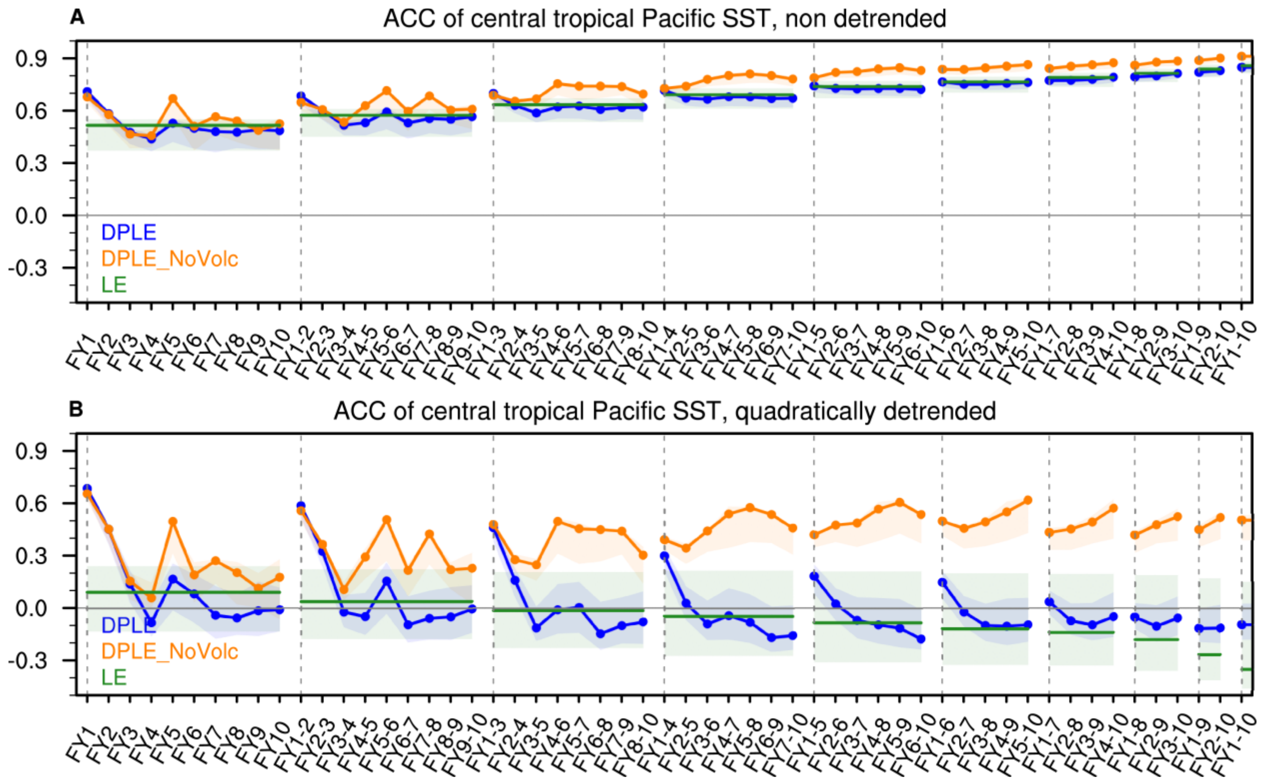

Fig. S4. As in Fig. 1J and Fig. S3J but for the central tropical Pacific. ACC of (**A**) non-detrended and (**B**) quadratically detrended central tropical Pacific SST (20°S–20°N, 160°E–140°W) as a function of lead time intervals corresponding to 1-yr–10-yr averages for DPLE (blue curves), DPLE_NoVolc (orange curves), and LE (green lines). Colored shading denotes the 10th–90th percentile range of ACC based on 5,000 bootstrapped ensemble means from randomly sampled 10-member ensembles, while the curves show ACC for the full ensembles (40, 10, and 40 members for DPLE, DPLE_NoVolc, and LE, respectively).

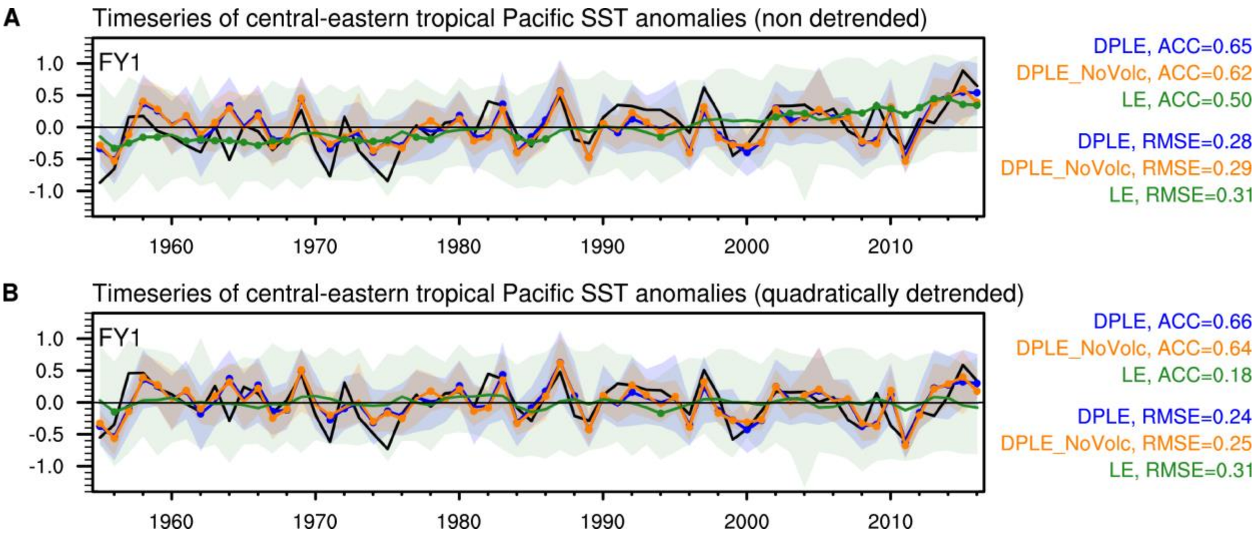

Fig. S5. Timeseries of (**A**) non-detrended and (**B**) quadratically detrended SST (°C) anomalies (curves) over the central-eastern tropical Pacific (20°S–20°N; 160°E–80°W) in observations (black) and ensemble-mean forecasts/simulations from DPLE (blue), DPLE_NoVolc (orange), and LE (green) for FY1. Shading denotes the range (minimum to maximum) of 40, 10, and 40 members for DPLE, DPLE_NoVolc, and LE, respectively. The ACC and RMSE for the initialized forecasts and LE are indicated on the right of each panel.

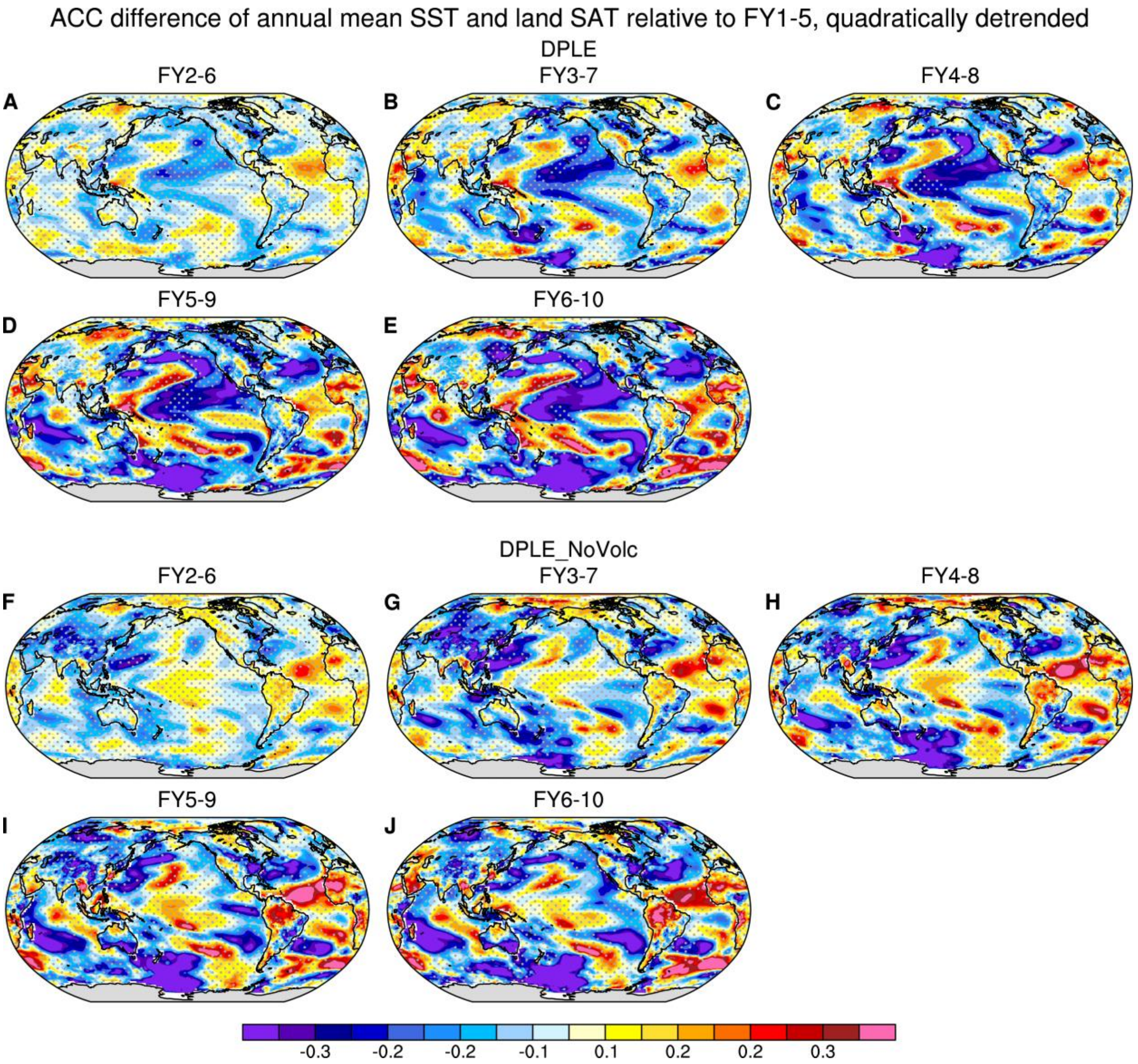

Fig. S6. ACC changes for quadratically detrended surface temperatures with lead times. ACC change for quadratically detrended annual SST and SAT over land at (**A**) FY2–6, (**B**) FY3–7, (**C**) FY4–8, (**D**) FY5–9, and (**E)** FY6–10 relative to the ACC at FY1–5 (see Fig. 1A) in DPLE. ACC change for quadratically detrended annual SST and SAT over land at (**F**) FY2–6, (**G**) FY3–7, (**H**) FY4–8, (**I**) FY5–9, and (**J**) FY6–10 relative to the ACC at FY1–5 (see Fig. 1D) in DPLE_NoVolc.

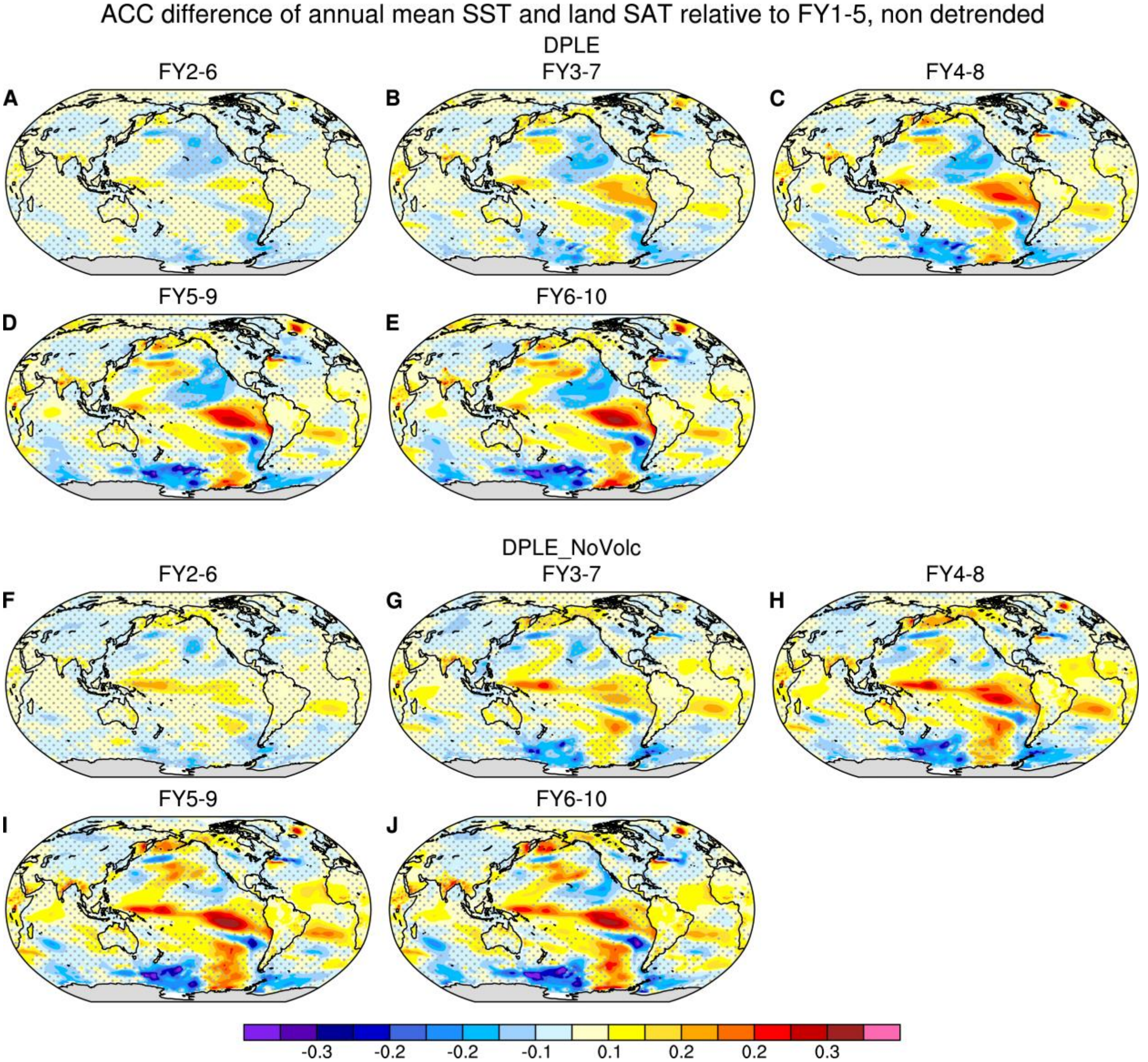

Fig. S7. ACC changes for surface temperatures with lead times. As in Fig. S6 but for non-detrended data. ACC change for annual SST and SAT over land at (**A**) FY2–6, (**B**) FY3–7, (**C**) FY4–8, (**D**) FY5–9, and (**E**) FY6–10 relative to the ACC at FY1–5 (see Fig. S3A) in DPLE. ACC change for annual SST and SAT over land at (**F**) FY2–6, (**G**) FY3–7, (**H**) FY4–8, (**I**) FY5–9, and (**J**) FY6–10 relative to the ACC at FY1–5 (see Fig. S3D) in DPLE_NoVolc.

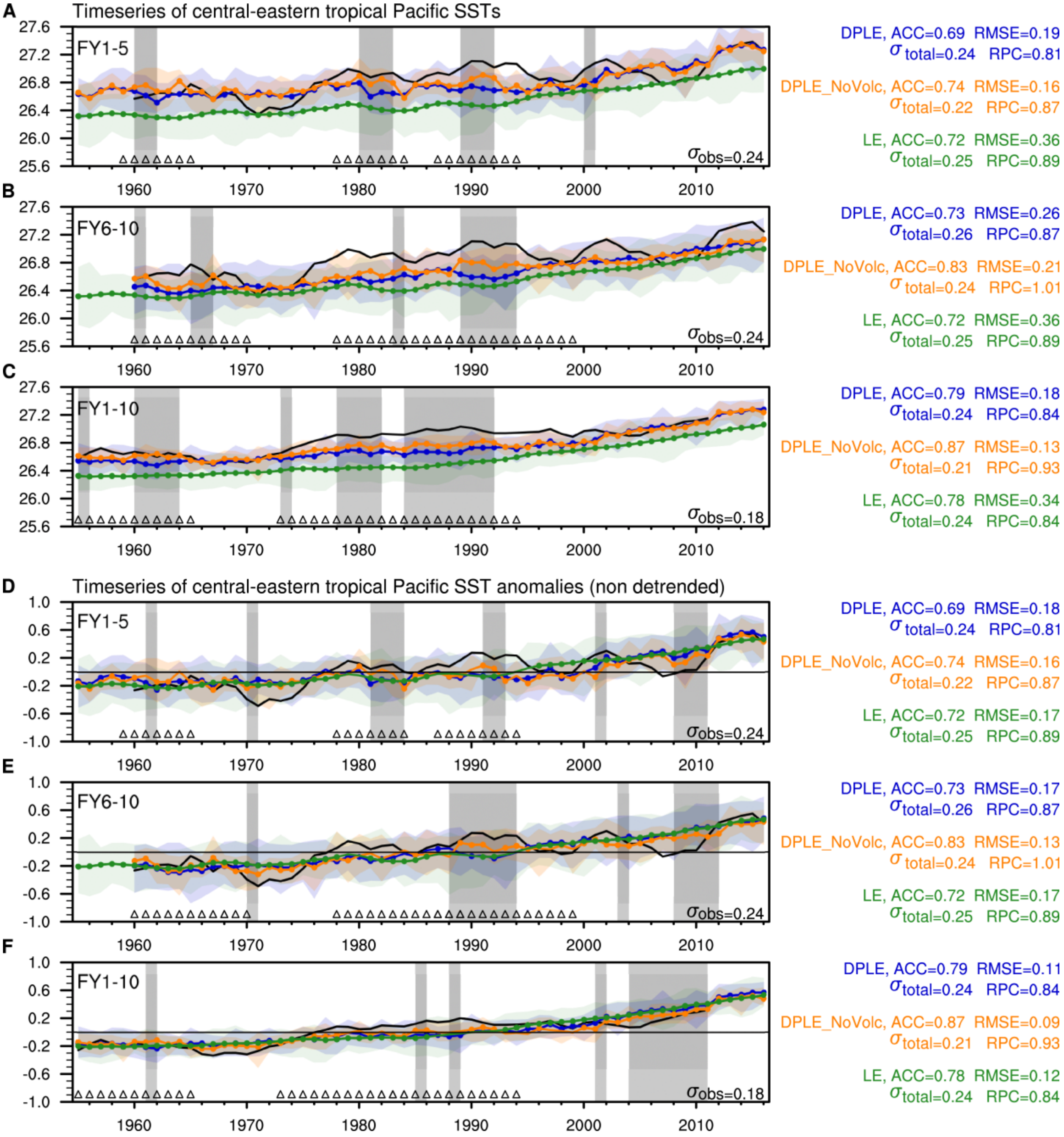

Fig. S8. Tropical Pacific SSTs or SST anomalies following major tropical volcanic eruptions. As in Fig. 2**A-C** in the main text but for the (**A-C**) original SSTs and (**D-F**) de-drifted but non-detrended SST anomalies over the central-eastern tropical Pacific (20°S–20°N; 160°E–80°W) in observations (black) and ensemble-mean forecasts from DPLE (blue), DPLE_NoVolc (red), and LE (green) for (**A** and **D**) FY1–5; (**B** and **E**) FY6–10, and (**C** and **F**) FY1–10. Shading denotes the range (minimum to maximum) of 40, 10, and 40 members for DPLE, DPLE_NoVolc, and LE, respectively. The year value in the x-axis denotes the start year of any 5-year or 10-year averaging window (e.g., 1960 represents 5-yr average anomalies spanning 1960–1964 corresponding to Nov 1959 initialization for FY1–5 in **A** and Nov 1954 initialization for FY6–10 in **B**). The triangles at the bottom of **A-C** denote the forecast ensembles that coincide with any of the three strong volcanic

eruptions starting in 1963, 1982, and 1991. The colored dots in **D-F** on curves denote the ensemble mean anomalies that are significant at the 90% confidence level, and the gray blocks highlight time periods when the ensemble mean difference between DPLE and DPLE_NoVolc is significant at the 90% confidence level based on bootstrapping across ensemble members (see Materials and Methods). The standard deviation of observed SST timeseries is indicated at the bottom right of each panel. The ACC, RMSE, standard deviation of total variability, and RPC for the initialized forecasts and LE are indicated on the right of each panel.

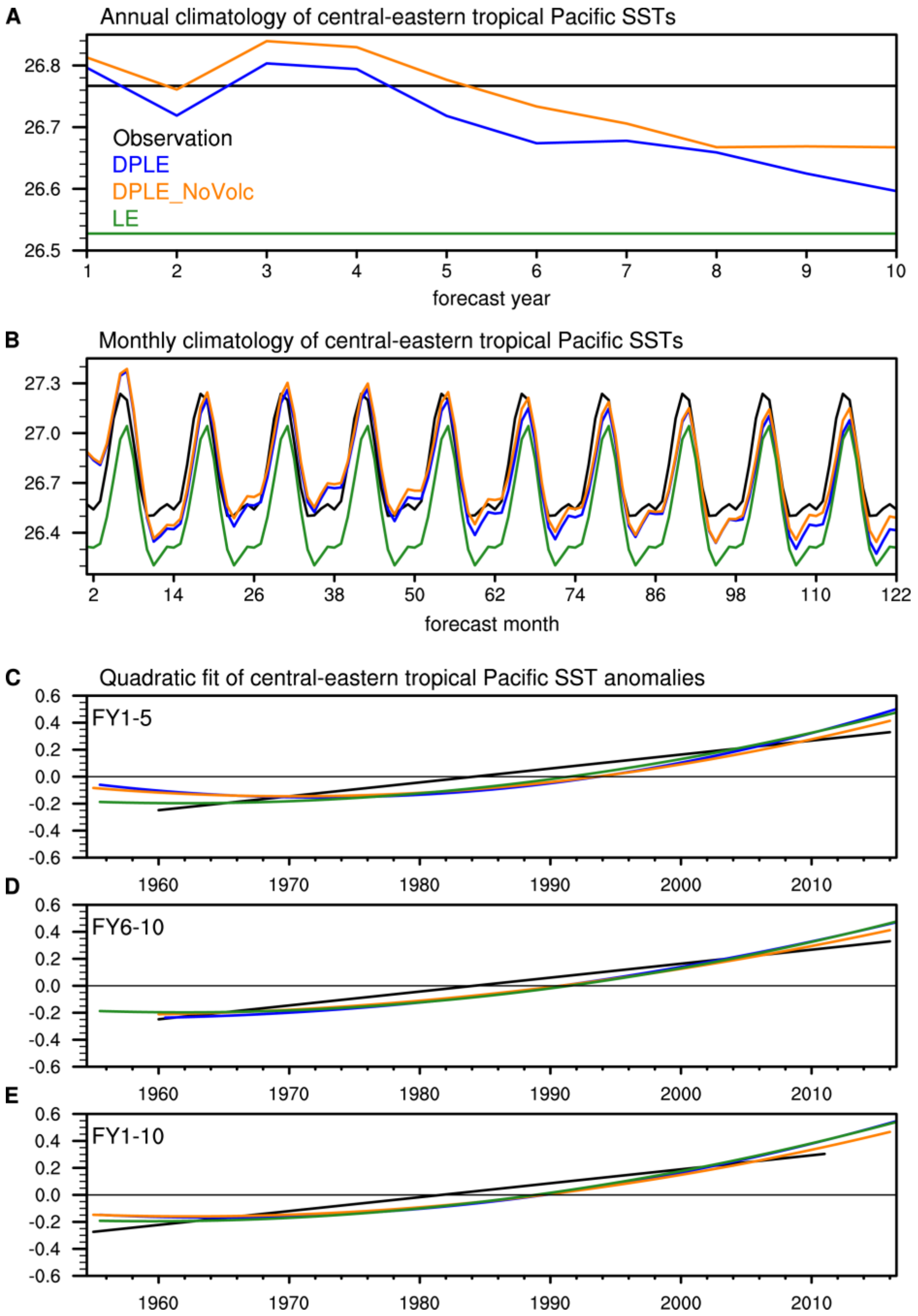

Fig. S9. Tropical Pacific SST climatology and trend. The (**A**) annual and (**B**) monthly climatology of SSTs (°C) over the central-eastern tropical Pacific (20°S–20°N; 160°E–80°W) in observations (black line), DPLE (blue curve), DPLE_NoVolc (orange curve), and LE (green curve). The DPLE and DPLE_NoVolc curves are a function of (**A**) forecast year 1–10 and (**B**) forecast month 1–122. Least squares quadratic fit of timeseries of SST anomalies (°C) in the central-eastern tropical Pacific in observations (black curve), DPLE (blue curve), DPLE_NoVolc (orange curve), and LE (green curve) for (**C**) FY1–5, (**D**) FY6–10, and (**E**) FY1–10.

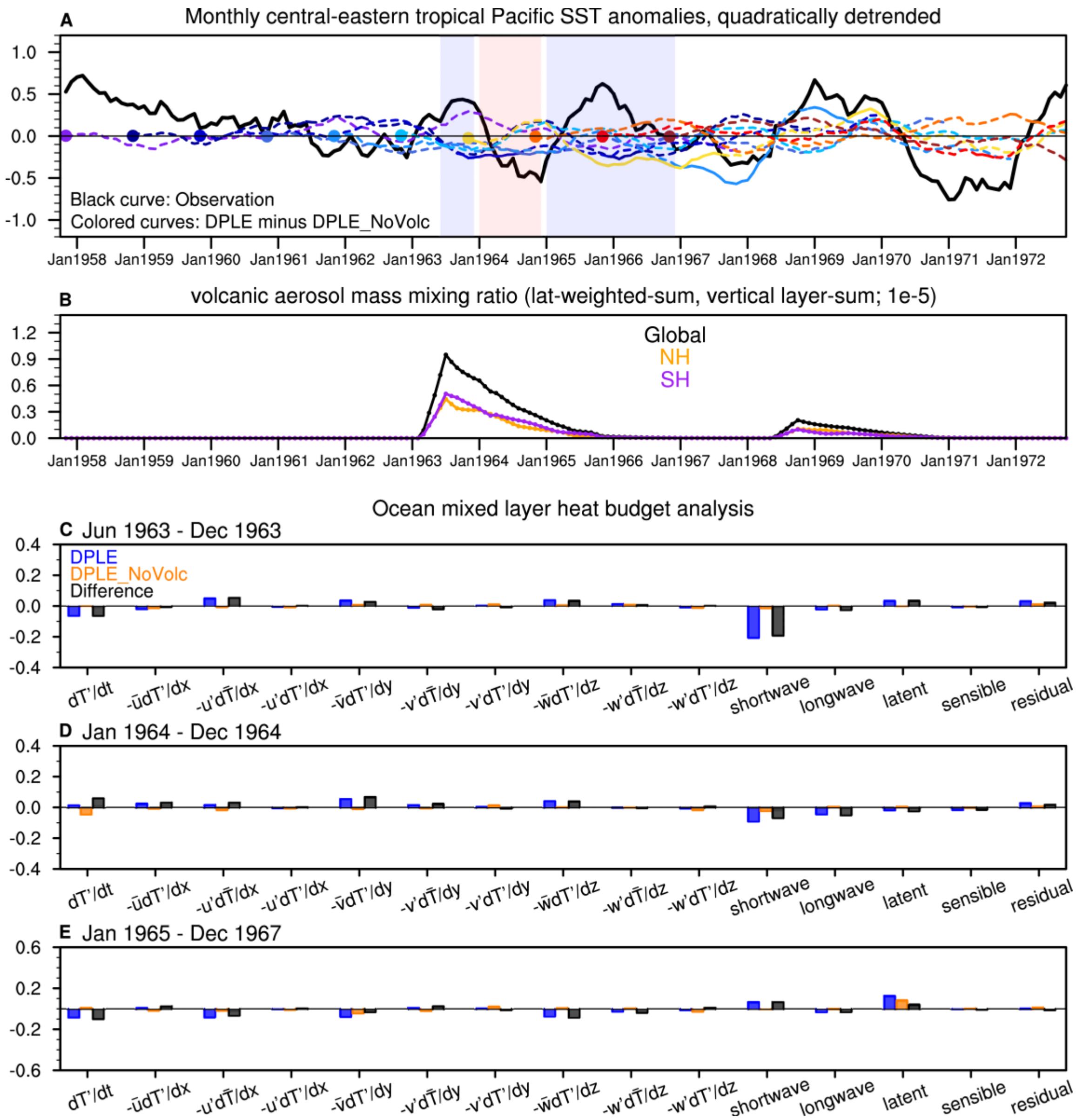

Fig. S10. Central-eastern tropical Pacific SST response to the Agung volcanic eruption in CESM1. As in Fig. 4 in the main text but for the Agung eruption. (**A**) Timeseries of monthly detrended SST anomalies over the central-eastern tropical Pacific (20°S–20°N, 160°E–80°W) in observations (thick black curve) and the ensemble-mean difference between DPLE and DPLE_NoVolc (colored curves) in the forecasts from 10 initial dates [Nov 1 1957–Nov 1 1966 denoted by colored dots; the solid portion of the curves indicates differences that are significant at the 90% confidence level based on bootstrapping across ensemble members (Materials and Methods)]. The light blue and red shaded blocks denote the phases of negative and positive SST tendencies, respectively. (**B**) Monthly time series of volcanic aerosol mass mixing ratios for the globe (black curve), the Northern Hemisphere (orange curve), and the Southern Hemisphere (purple curve). (**C-E**) ensemble-mean ocean mixed layer heat balance terms (see Materials and Methods) averaged in the same region and integrated over three stages (Jun 1963–Dec 1963, Jan 1964–Dec 1964, and Jan 1965–Dec 1966) composited for the 6 ensembles initialized in Nov 1957–Nov 1962 in DPLE (blue), DPLE_NoVolc (orange), and their difference (black).

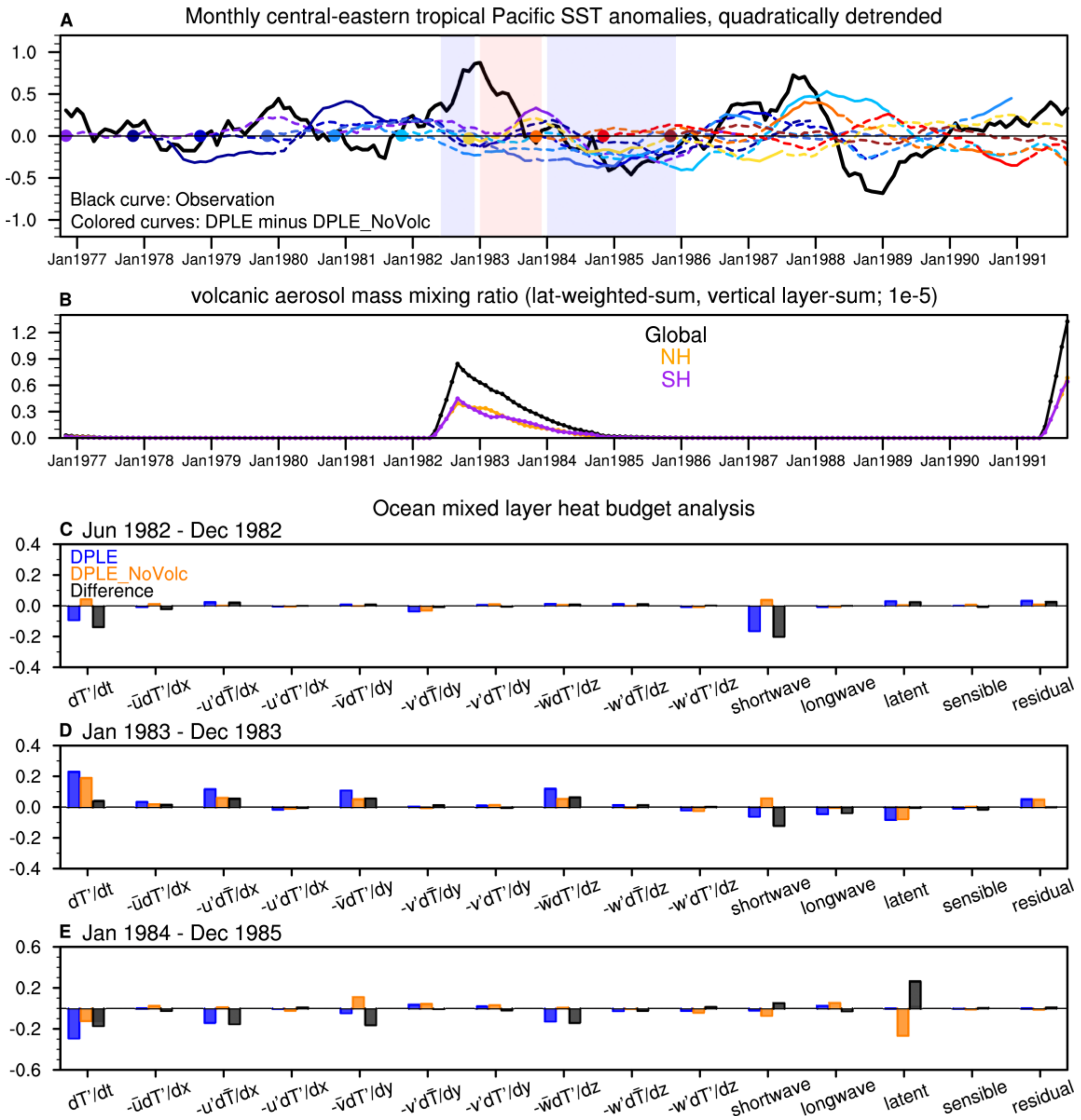

Fig. S11. Central-eastern tropical Pacific SST response to the El Chichón volcanic eruption in CESM1. As in Fig. 4 in the main text but for the El Chichón eruption. (**A**) Timeseries of monthly detrended SST anomalies over the central-eastern tropical Pacific (20°S–20°N, 160°E–80°W) in observations (thick black curve) and the ensemble-mean difference between DPLE and DPLE_NoVolc (colored curves) in the forecasts from 10 initial dates [Nov 1 1976–Nov 1 1985 denoted by colored dots; the solid portion of curves indicates the difference that is significant at the 90% confidence level based on bootstrapping across ensemble members (Materials and Methods)]. The light blue and red shaded blocks denote the phases of negative and positive SST tendencies, respectively. (**B**) Monthly time series of volcanic aerosol mass mixing ratios for the globe (black curve), the Northern Hemisphere (orange curve), and the Southern Hemisphere (purple curve). (**C-E**), ensemble-mean ocean mixed layer heat balance terms (see Materials and Methods) averaged in the same region and integrated over three stages (Jun 1982–Dec 1982, Jan 1983–Dec 1983, and Jan 1984–Dec 1985) composited for the 6 ensembles initialized in Nov 1976–Nov 1981 in DPLE (blue), DPLE_NoVolc (orange), and their difference (black).

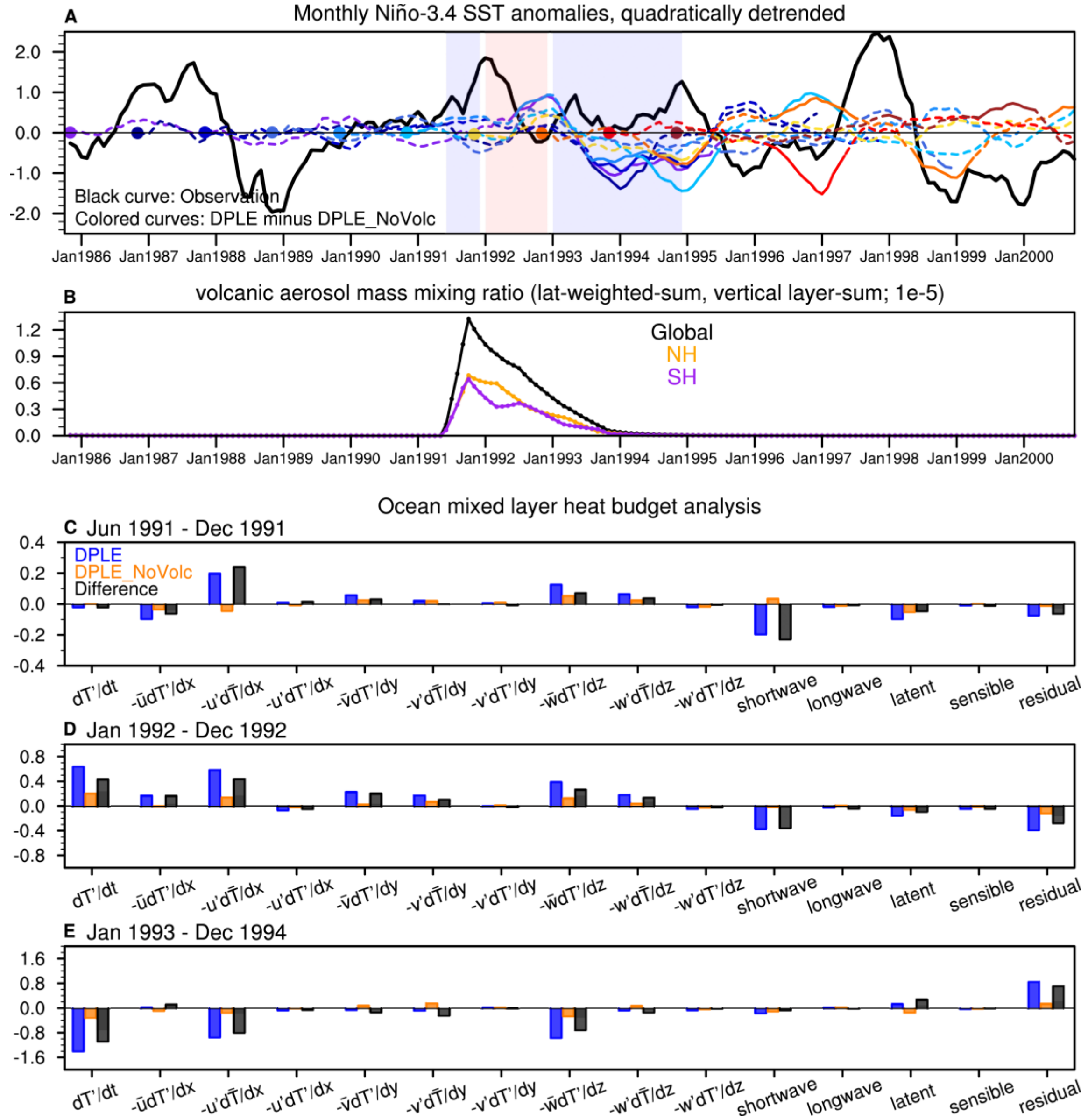

Fig. S12. ENSO SST response to the Pinatubo volcanic eruption in CESM1. As in Fig. 4 in the main text but for the Niño-3.4 region. (**A**) Timeseries of monthly detrended SST anomalies over the Niño-3.4 region (5°S–5°N, 170°W–120°W) in observations (thick black curve) and the ensemble-mean difference between DPLE and DPLE_NoVolc (colored curves) in the forecasts from 10 initial dates [Nov 1 1985–Nov 1 1994 denoted by colored dots; the solid portion of curves indicates the difference that is significant at the 90% confidence level based on bootstrapping across ensemble members (Materials and Methods)]. The light blue and red shaded blocks denote the phases of negative and positive SST tendencies, respectively. (**B**) Monthly time series of volcanic aerosol mass mixing ratios for the globe (black curve), the Northern Hemisphere (orange curve), and the Southern Hemisphere (purple curve). (**C-E**), ensemble-mean ocean mixed layer heat balance terms (see Materials and Methods) averaged in the same region and integrated over three stages (Jun 1991–Dec 1991, Jan 1992–Dec 1992, and Jan 1993–Dec 1994) composited for the 6 ensembles initialized in Nov 1985–Nov 1990 in DPLE (blue), DPLE_NoVolc (orange), and their difference (black).

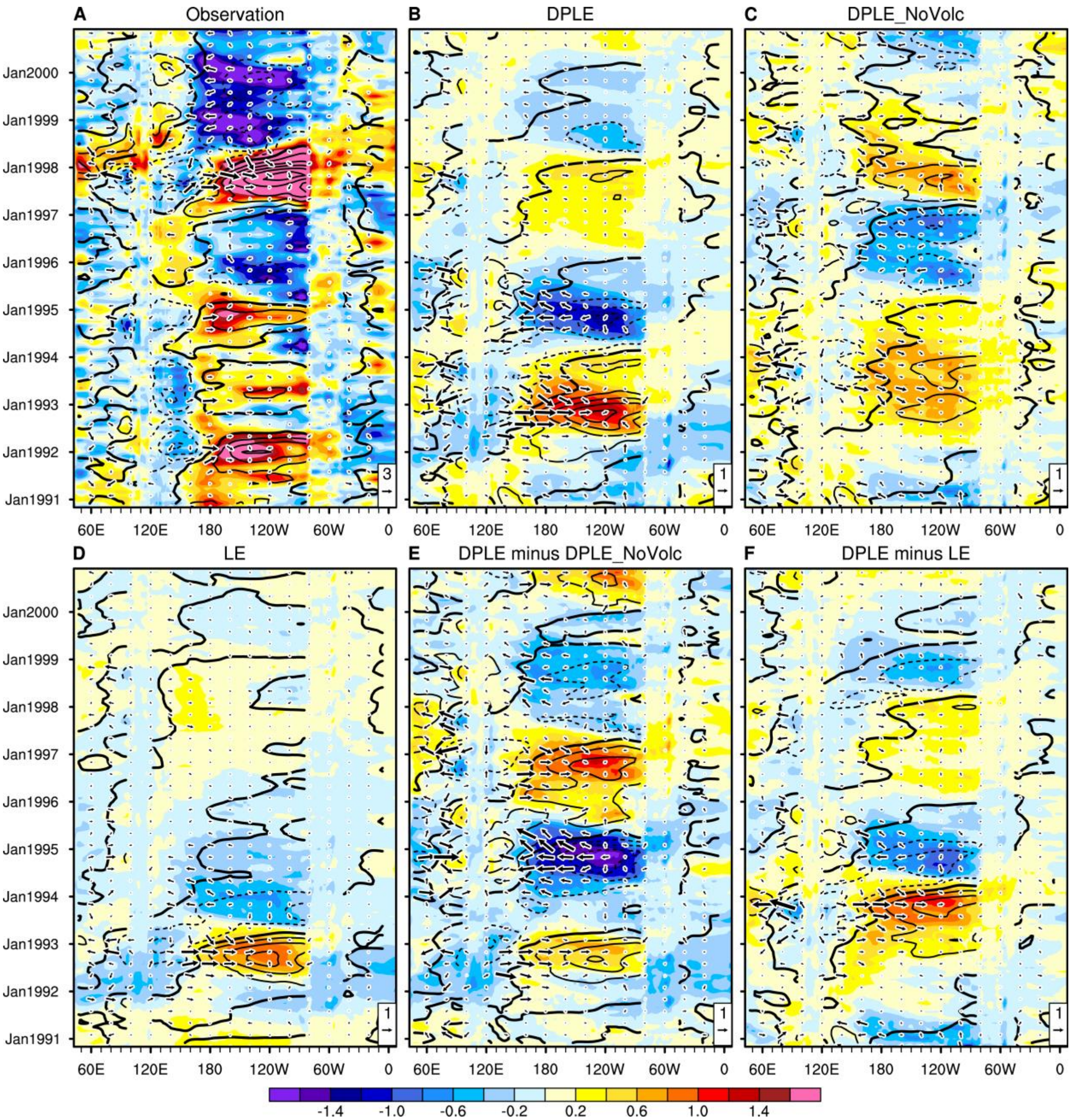

Fig. S13. Equatorial dynamical response to the Pinatubo eruption. Longitude-time sections of detrended SST (°C; color shading), surface wind (m s^{-1}; vectors), and sea surface height (SSH; contours at intervals of 6 cm for **A** and of 2 cm for **B-F**; zero contours thickened and negative contours dashed) anomalies along the equator (3°S–3°N) during Nov 1991–Dec 2000 in (**A**) observation, and ensemble-mean simulations from (**B**) DPLE, (**C**) DPLE_NoVolc, (**D**) LE, (**E**) the difference between DPLE and DPLE_NoVolc, and (**F**) the difference between DPLE and LE. The surface wind anomalies are shown every 3 months starting at November (Nov, Feb, May, and Aug) for better visualization.

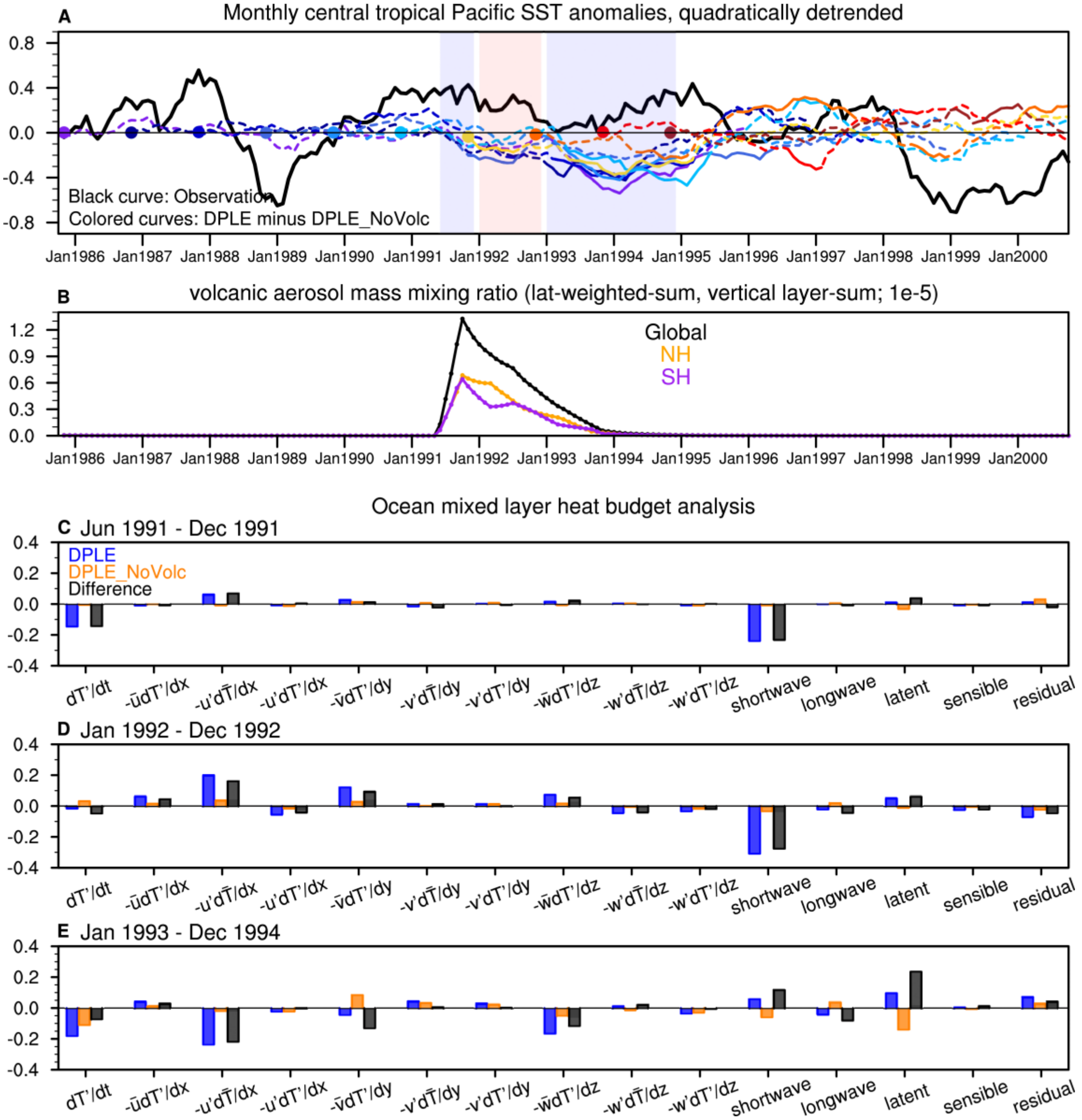

Fig. S14. Central tropical Pacific SST response to the Pinatubo volcanic eruption in CESM1. As in Fig. 4 in the main text but for the central tropical Pacific region. (**A**) Timeseries of monthly detrended SST anomalies over the central tropical Pacific region (20°S–20°N, 160°E–140°W) in observations (thick black curve) and the ensemble-mean difference between DPLE and DPLE_NoVolc (colored curves) in the forecasts from 10 initial dates [Nov 1 1985–Nov 1 1994 denoted by colored dots; the solid portion of curves indicates the difference that is significant at the 90% confidence level based on bootstrapping across ensemble members (Materials and Methods)]. The light blue and red shaded blocks denote the phases of negative and positive SST tendencies, respectively. (**B**) Monthly time series of volcanic aerosol mass mixing ratios for the globe (black curve), the Northern Hemisphere (orange curve), and the Southern Hemisphere (purple curve). (**C-E**) ensemble-mean ocean mixed layer heat balance terms (see Materials and Methods) averaged in the same region and integrated over three stages (Jun 1991–Dec 1991, Jan 1992–Dec 1992, and Jan 1993–Dec 1994) composited for the 6 ensembles initialized in Nov 1985–Nov 1990 in DPLE (blue), DPLE_NoVolc (orange), and their difference (black).

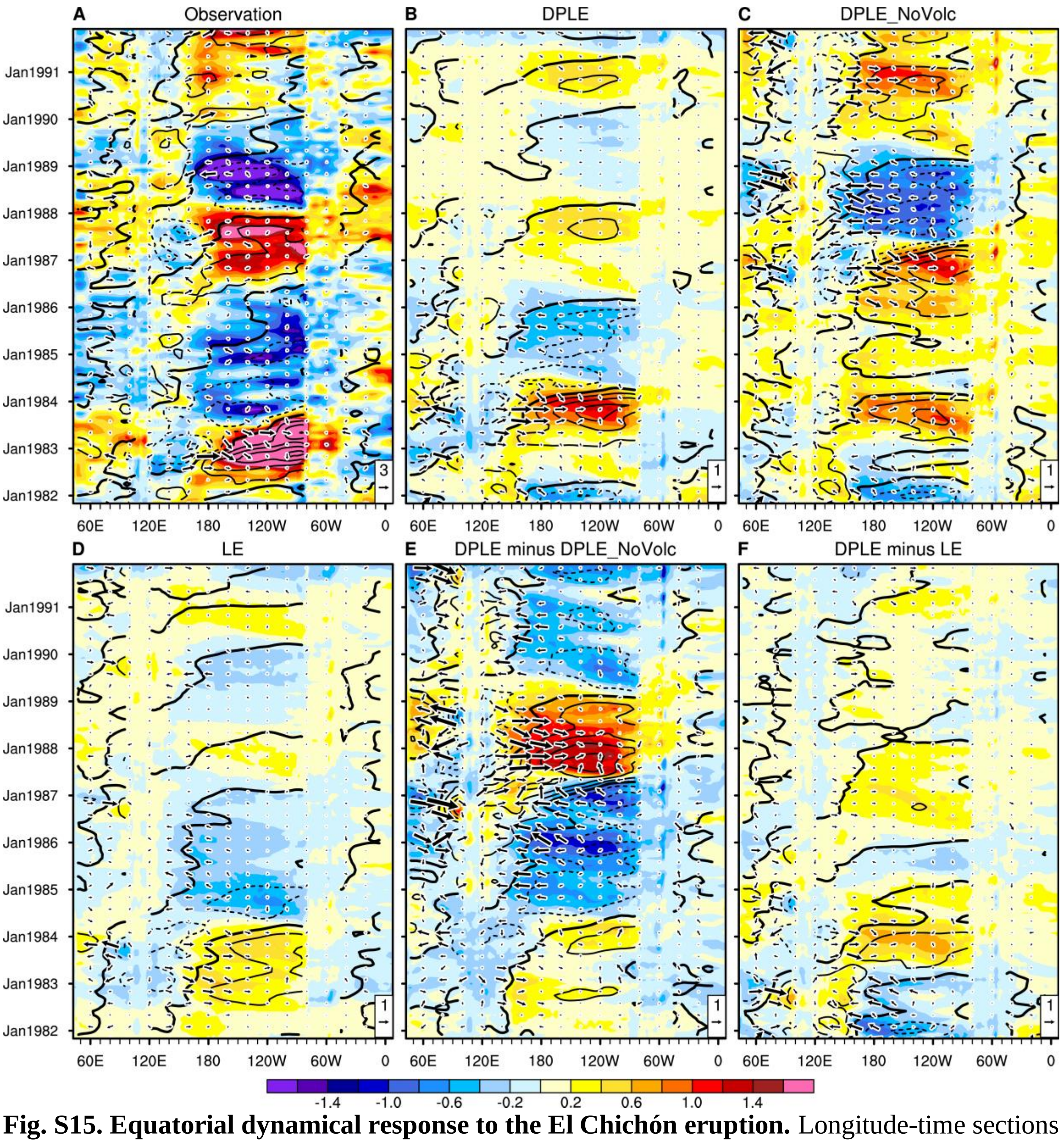

Fig. S15. Equatorial dynamical response to the El Chichón eruption. Longitude-time sections of detrended SST (°C; color shading), surface wind (m s^{-1}; vectors), and sea surface height (SSH; contours at intervals of 6 cm for **A** and of 2 cm for **B-F**; zero contours thickened and negative contours dashed) anomalies along the equator (3°S–3°N) during Nov 1981–Dec 1991 in (**A**) observation, and ensemble-mean simulations from (**B**) DPLE, (**C**) DPLE_NoVolc, (**D**) LE, (**E**) the difference between DPLE and DPLE_NoVolc, and (**F**) the difference between DPLE and LE. The surface wind anomalies are shown every 3 months starting at November (Nov, Feb, May, and Aug) for better visualization.

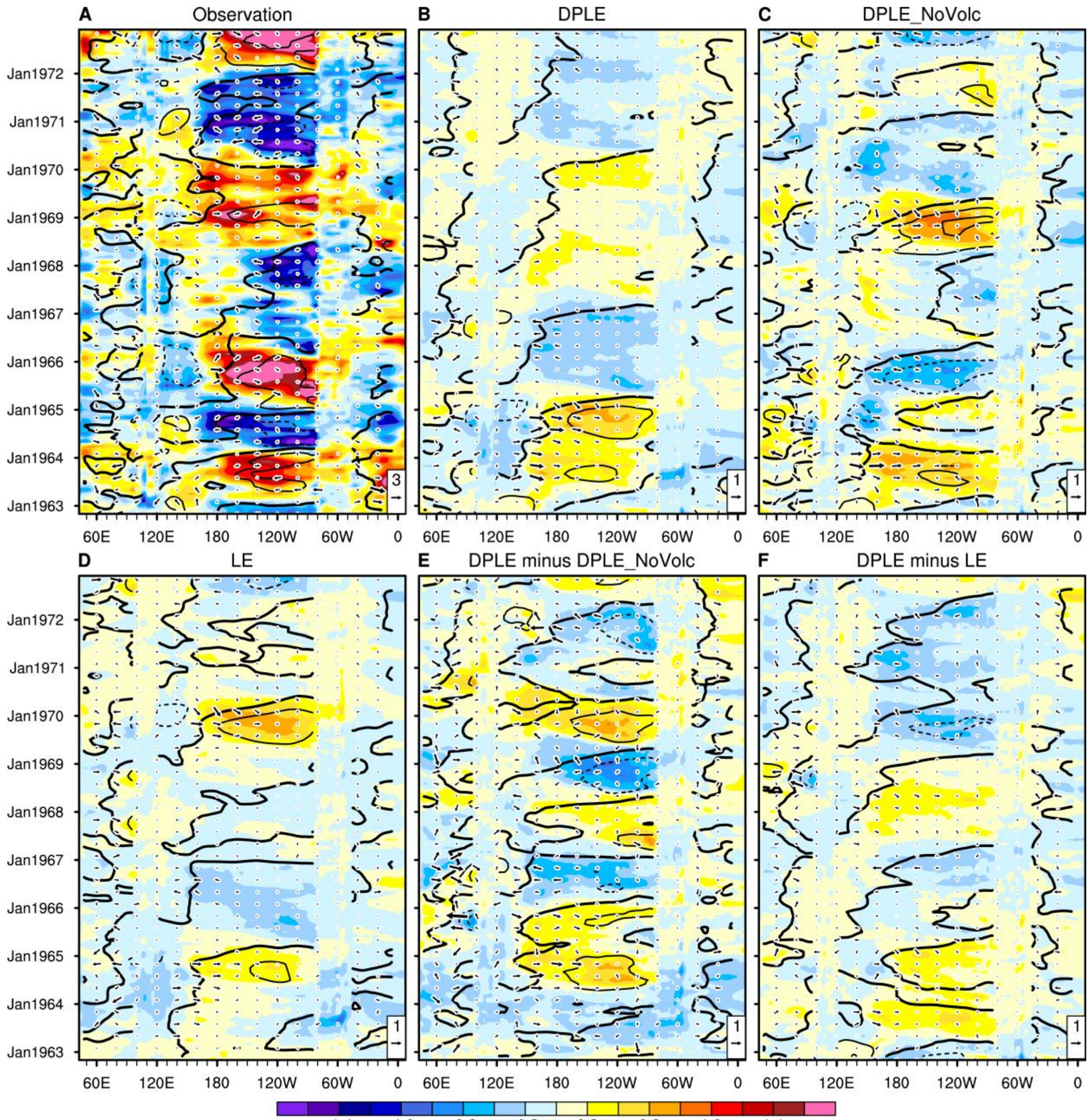

Fig. S16. Equatorial dynamical response to the Agung eruption. Longitude-time sections of detrended SST (°C; color shading), surface wind (m s^{-1}; vectors), and sea surface height (SSH; contours at intervals of 6 cm for **A** and of 2 cm for **B-F**; zero contours thickened and negative contours dashed) anomalies along the equator (3°S–3°N) during Nov 1962–Dec 1972 in (**A**) observation, and ensemble-mean simulations from (**B**) DPLE, (**C**) DPLE_NoVolc, (**D**) LE, (**E**) the difference between DPLE and DPLE_NoVolc, and (**F**) the difference between DPLE and LE. The surface wind anomalies are shown every 3 months starting at November (Nov, Feb, May, and Aug) for better visualization.
